# Quality of reporting in AI cardiac MRI segmentation studies – A systematic review and recommendations for future studies

**DOI:** 10.3389/fcvm.2022.956811

**Published:** 2022-07-15

**Authors:** Samer Alabed, Ahmed Maiter, Mahan Salehi, Aqeeb Mahmood, Sonali Daniel, Sam Jenkins, Marcus Goodlad, Michael Sharkey, Michail Mamalakis, Vera Rakocevic, Krit Dwivedi, Hosamadin Assadi, Jim M. Wild, Haiping Lu, Declan P. O’Regan, Rob J. van der Geest, Pankaj Garg, Andrew J. Swift

**Affiliations:** ^1^Department of Infection, Immunity and Cardiovascular Disease, The University of Sheffield, Sheffield, United Kingdom; ^2^Department of Clinical Radiology, Sheffield Teaching Hospitals, Sheffield, United Kingdom; ^3^INSIGNEO, Institute for *in silico* Medicine, The University of Sheffield, Sheffield, United Kingdom; ^4^Medical School, The University of Sheffield, Sheffield, United Kingdom; ^5^University of East Anglia, Norwich Medical School, Norwich, United Kingdom; ^6^Department of Computer Science, The University of Sheffield, Sheffield, United Kingdom; ^7^MRC London Institute of Medical Sciences, Imperial College London, London, United Kingdom; ^8^Leiden University Medical Center, Leiden, Netherlands

**Keywords:** artificial intelligence, machine learning, cardiac MRI, segmentation, systematic review, quality, reporting

## Abstract

**Background:**

There has been a rapid increase in the number of Artificial Intelligence (AI) studies of cardiac MRI (CMR) segmentation aiming to automate image analysis. However, advancement and clinical translation in this field depend on researchers presenting their work in a transparent and reproducible manner. This systematic review aimed to evaluate the quality of reporting in AI studies involving CMR segmentation.

**Methods:**

MEDLINE and EMBASE were searched for AI CMR segmentation studies in April 2022. Any fully automated AI method for segmentation of cardiac chambers, myocardium or scar on CMR was considered for inclusion. For each study, compliance with the Checklist for Artificial Intelligence in Medical Imaging (CLAIM) was assessed. The CLAIM criteria were grouped into study, dataset, model and performance description domains.

**Results:**

209 studies published between 2012 and 2022 were included in the analysis. Studies were mainly published in technical journals (58%), with the majority (57%) published since 2019. Studies were from 37 different countries, with most from China (26%), the United States (18%) and the United Kingdom (11%). Short axis CMR images were most frequently used (70%), with the left ventricle the most commonly segmented cardiac structure (49%). Median compliance of studies with CLAIM was 67% (IQR 59–73%). Median compliance was highest for the model description domain (100%, IQR 80–100%) and lower for the study (71%, IQR 63–86%), dataset (63%, IQR 50–67%) and performance (60%, IQR 50–70%) description domains.

**Conclusion:**

This systematic review highlights important gaps in the literature of CMR studies using AI. We identified key items missing—most strikingly poor description of patients included in the training and validation of AI models and inadequate model failure analysis—that limit the transparency, reproducibility and hence validity of published AI studies. This review may support closer adherence to established frameworks for reporting standards and presents recommendations for improving the quality of reporting in this field.

**Systematic Review Registration:**

[www.crd.york.ac.uk/prospero/], identifier [CRD42022279214].

## Introduction

Cardiac MRI (CMR) is the gold standard for non-invasive assessment of cardiac structures. Quantitative measurement of cardiac volumes can be achieved with CMR and relies on accurate segmentation of structures on CMR images. Manual segmentation is routinely performed by cardiac imaging experts but suffers from a number of drawbacks. In addition to being laborious and time-intensive, manual segmentation is operator-dependent, potentially impacting interobserver agreement. As the demand for cardiac imaging continues to grow and outpaces the supply of trained readers, there is an increasing need for automation (1, 2).

Artificial intelligence (AI) is changing medical imaging through the automation of complex and repetitive tasks, including the segmentation of anatomical structures (3). Machine learning is a subfield of AI that is commonly used for image analysis and processing in medical applications. Machine learning algorithms learn by experience, typically in a supervised manner: the algorithm is trained on labeled data, such as a set of manually segmented CMR images, where the manual segmentation provides the reference standard or ground truth. The algorithm identifies discriminative features and patterns in this image data, which are incorporated to generate a model that can perform the task—such as segmentation of the cardiac chambers—on new unlabeled data without the need for explicit programming. Machine learning itself encompasses a diverse range of techniques, including deep learning, which can be applied to the segmentation of structures in imaging (4).

A growing number of studies have reported the use of AI methods for segmentation in CMR. The manner in which these studies are reported is important. Transparent reporting of methods and results facilitates reproducibility and allows proper evaluation of validity. Equally, a consistent standard of reporting aids comparison between studies and may improve accessibility of the literature, which may be of particular benefit in a rapidly expanding field such as AI. The need for consistency in reporting medical research is well recognized and reflected in various guidelines and checklists for different study types. The Checklist for Artificial Intelligence in Medical Imaging (CLAIM), (5) has adopted the validated and widely used Standards for Reporting of Diagnostic Accuracy Studies (STARD) guidelines and incorporated domains specific to AI studies, including detailed descriptions of data sources, model design and performance evaluation. This systematic review aimed to evaluate the quality of reporting of studies involving AI CMR segmentation by assessing compliance with CLAIM.

## Materials and methods

The study protocol was registered with The International Prospective Register of Systematic Reviews (PROSPERO; registry number CRD42022279214). The study was undertaken and is presented in accordance with the Preferred Reporting Items for Systematic reviews and Meta-Analyses (PRISMA) guidelines (6). No ethical approval was required.

### Inclusion and exclusion criteria

**FIGURE 1:**
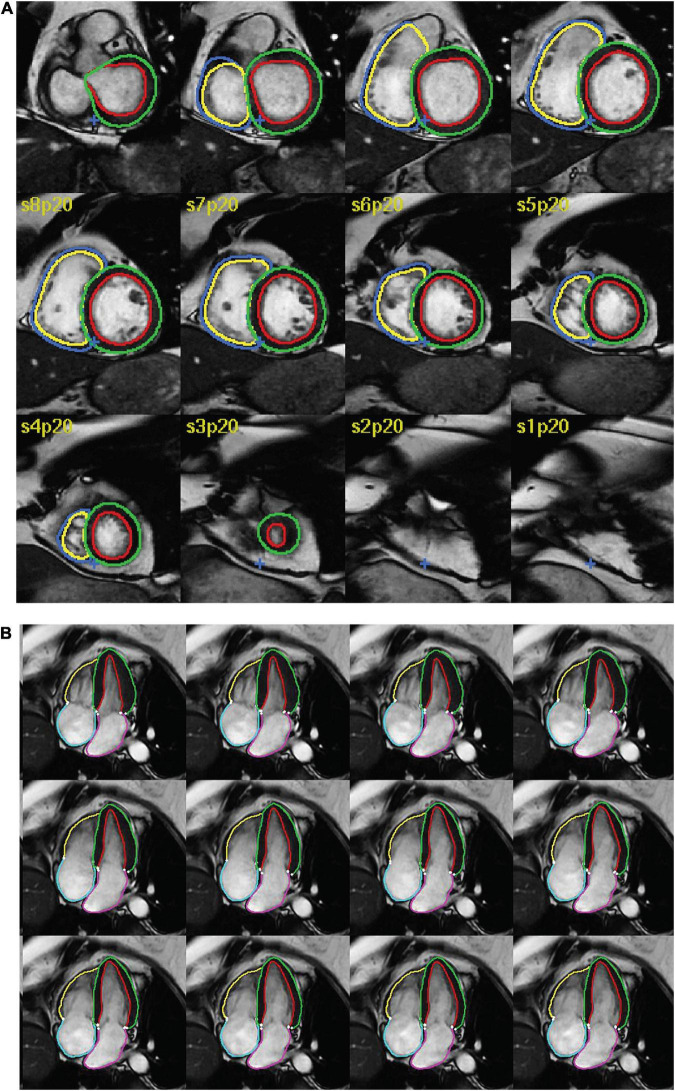
Examples of AI cardiac MRI segmentation. Examples of automatic **(A)** biventricular and **(B)** four-chamber segmentation. The colored contours in green and red show the left ventricular epi- and endocardium, respectively. The contours in dark blue and yellow show the right ventricular epi- and endo- cardium, respectively. The pink and turquoise contours outline the left and right atria, respectively.

Studies reporting the use of AI for segmentation of structures in CMR were considered for inclusion. Studies were deemed eligible if they reported: (1) any type of fully automated AI method (including machine learning, deep learning and neural networks), (2) segmentation of cardiac chambers, myocardium or scar tissue and (3) use of adult human CMR images, regardless of acquisition methods (such as use of intravenous contrast), parameters, post-processing methods and software. Exclusion criteria were as follows: absence of a newly developed segmentation model (e.g., studies assessing existing methods), use of semi-automated AI methods (where the segmentation process required manual input), multiorgan segmentation, combined segmentation of multiple imaging modalities (e.g., CMR and CT), segmentation of cardiac vessels (e.g., aorta, pulmonary artery, coronary arteries) or pericardial tissue, use of non-human or *ex vivo* images, and conference publications. Figure 1 shows an example of automatic biventricular (7) (Figure 1A) and four-chamber (8) (Figure 1B) segmentation on CMR.

### Search method

The MEDLINE and EMBASE databases were searched for relevant studies on April 20 2022. The search strategy is outlined in the Supplementary Material. Non-English language publications were excluded.

### Study selection

**FIGURE 2:**
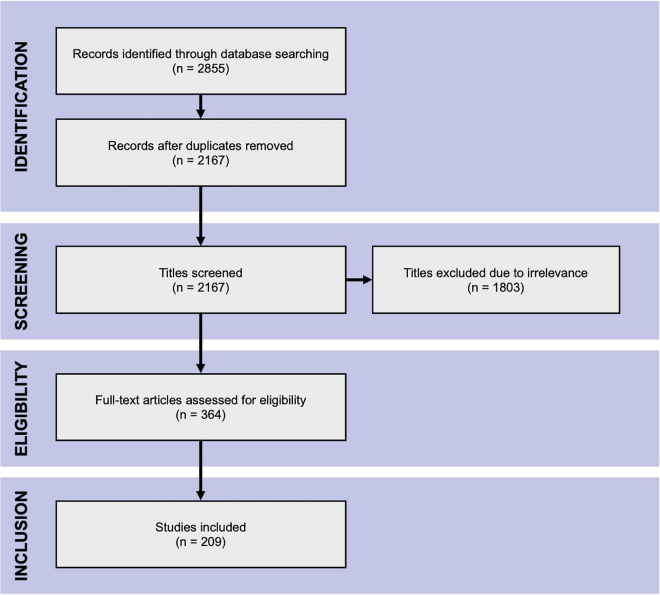
PRISMA flow chart. The Preferred Reporting Items for Systematic Reviews and Meta-Analyses flow chart of literature search.

Figure 2 indicates the flow of study identification and inclusion. Duplicate studies were removed following the initial database search. The titles and abstracts of the remaining studies were screened for relevance. The full texts of all potentially relevant studies were retrieved and assessed for eligibility against the inclusion and exclusion criteria. Conference abstracts and studies lacking sufficient information for evaluation were excluded at this point. Screening was performed independently by (SA) and by (SD, AM2, MS2) and full texts were assessed for eligibility by SA, AM1 and MS, with SA acting as an arbitrator.

### Data extraction

Three authors extracted data from the included studies (SA, AM1, MS1) according to a standardized checklist. Half of the included studies were also evaluated independently by an additional five authors (SD, AM2, SJ, MG, HA) for the purpose of quality control. All discrepancies were resolved with discussion, with SA acting as an arbitrator, and the final extracted data confirmed. Descriptive information about each study was recorded, including publication details (type, source, country, year), data used (type of data set, type of CMR image, segmented structures) and AI model (validation and performance evaluation methods). The studies were assessed for compliance against the 42 criteria of CLAIM, which were grouped into four domains: study description (9 criteria), dataset description (17 criteria), model description (6 criteria) and model performance (10 criteria). Supplementary Table 2 indicates all CLAIM criteria and their assignment to the domains. For each criterion, compliance was marked as yes, no or not applicable (N/A). Studies deemed N/A were excluded when evaluating the proportion of studies compliant with CLAIM criteria. For studies using solely public datasets, the following criteria were marked as N/A, as they can be considered implicit in the use of publicly available data sources: retrospective or prospective study, source of ground truth annotations, annotation tools, de-identification methods and inter- and intra-rater variability. Additionally, the following criteria were marked as N/A for all studies: rationale for choosing the reference standard (as manual expert contouring is the standard in the field) and registration number and name of registry. Descriptive data and the number of studies compliant with CLAIM criteria are presented as proportional values (%).

## Results

### Search results

The database search yielded 2,855 hits from which the title and abstract screening identified 364 relevant studies. The subsequent full-text assessment deemed 209 eligible for inclusion in the analysis (Figure 2).

### Included studies

**FIGURE 3:**
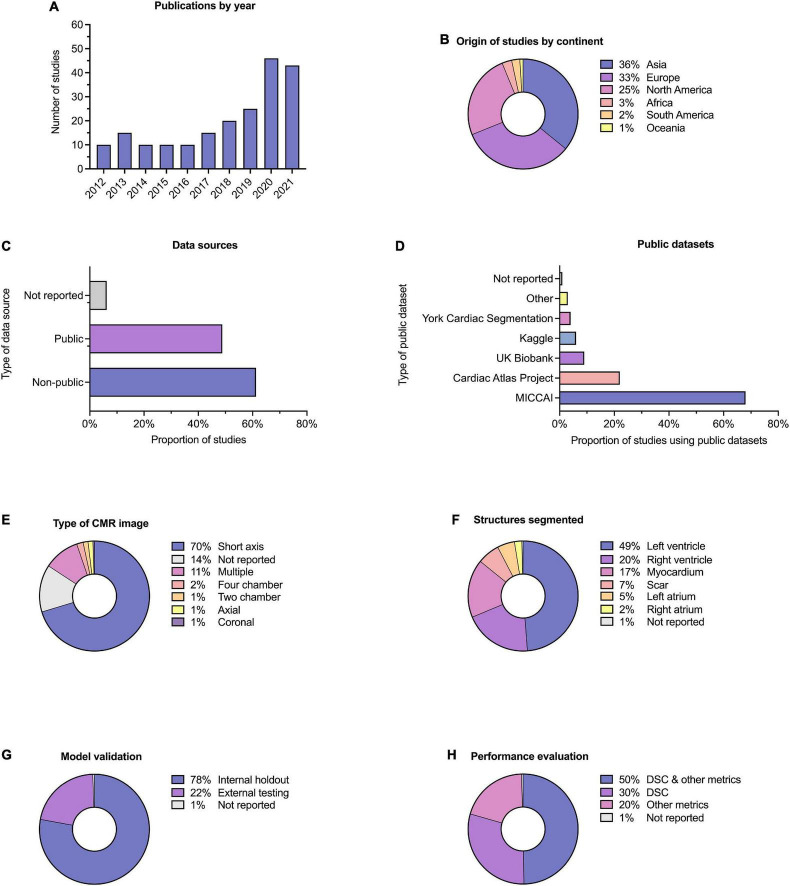
Descriptive information. Descriptive information for the 209 included studies. **(A)** Publication dates; five studies (2.4%) were included from early 2022 and are not indicated here. **(B)** Location of origin of studies. **(C)** Data sources; the proportion of studies which used public and non-public datasets is shown, with some studies having used multiple or combined datasets. **(D)** Public datasets used by studies, where relevant. **(E)** Type of CMR images used. **(F)** Cardiac structures segmented; some studies performed segmentation on multiple structures. **(G)** Method of model validation. **(H)** Method of model performance evaluation.

Descriptive information for all of the 209 included studies are provided in Supplementary Table 1. Selected metrics are highlighted in Figure 3. The majority of studies (57%) were published since 2019 (Figure 3A). Most studies were published in technical journals (58%), with a minority published in medical (31%) or hybrid (11%) journals. The studies were undertaken in 37 different countries (Figure 3B), with just over half coming from China (26%), the United States (18%) and the United Kingdom (11%).

Publicly available datasets were used in 49% of studies, and single or multicenter non-public datasets used in 61%, 17% of studies used multiple or combined datasets (including multiple public datasets and a combination of public and non-public datasets). A minority of studies (6%) did not report their data source (Figure 3C). Of the public datasets used, the majority (86%) had been made available through Medical Image Computing and Computer-Assisted Intervention (MICCAI) challenges or the Cardiac Atlas Project (9) (Figure 3D). Most studies reported the number of cases used (95%), with a range of 3–12,984 and a median of 78. Short axis CMR images were most frequently used (70%), while 14% of studies did not report the specific type of CMR image used for segmentation (Figure 3E). The left ventricle was the most commonly segmented structure, either alone or in combination (49%, Figure 3F). Segmentation of multiple structures was reported in 23% of studies.

Model validation was mostly reported using internal holdout methods (78%), such as cross-validation. A minority reported testing on external and mainly public datasets (22%, Figure 3G). The Dice similarity coefficient (DSC) was used to assess model performance in 79% of studies, either alone or in combination with other metrics such as the Hausdorff distance or the Jaccard index (Figure 3H). Few studies (10%) provided working links to publicly available code, with a further 1% indicating that code was available on request.

### Compliance with CLAIM

**FIGURE 4:**
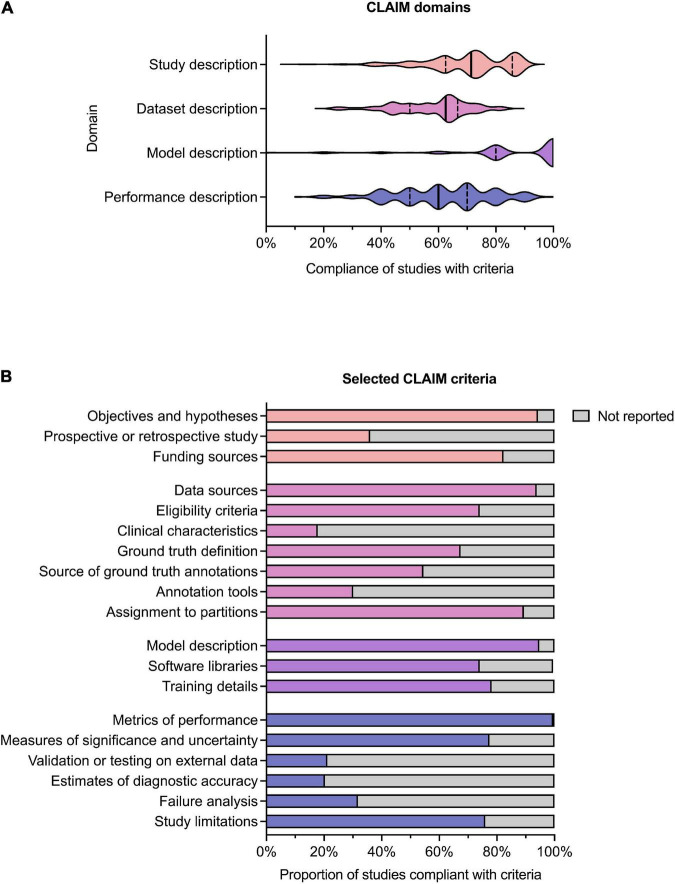
Compliance with CLAIM. **(A)** Violin plot showing compliance of the 209 included studies with the CLAIM criteria, grouped into domains of study, dataset, model and performance description. Median (*solid line*) and 1st and 3rd quartile (*dashed lines*) values are indicated. **(B)** Proportion of studies compliant with selected CLAIM criteria, grouped by domain (the titles of the individual criteria have been shortened for ease of reading).

Results for compliance with the domains and selected individual criteria of CLAIM are summarized in Figure 4. The complete results are presented in Supplementary Table 2. The median compliance of all studies with all 42 criteria of CLAIM was 67% (IQR 59–73%). Notable results excluding non-applicable criteria are as follows.

#### Study description

Median compliance with the study description domain was 71% (IQR 63–86%). Almost all studies clearly indicated the use of AI methods (91%) and their objectives (94%). Where non-public datasets were used, only a minority of studies (36%) indicated whether these were prospective or retrospective. No studies provided access to a full study protocol. Sources of funding were declared in 82% of studies.

#### Dataset description

Median compliance with the dataset description domain was 63% (IQR 50–67%), the lowest of the four domains. The source of the dataset was reported in most studies (94%). While most studies provided eligibility criteria for included cases (74%), few studies reported their demographic and clinical characteristics (18%) or indicated the flow of these cases (10%) in sufficient detail. Details regarding the calculation of the intended sample size (4%) and how missing data were handled (9%) were also infrequently reported. The definition of the ground truth reference standard was provided in 68% of studies. Where non-public datasets were used, the source of ground truth annotations and annotation tools were stated in 55% and 31% of studies respectively, with inter- and intra-rater variability reported in 42%. The majority of studies reported data preprocessing steps (94%), definitions of data elements (99.5%), how data were assigned to partitions (89%) and the level at which partitions were disjoint (87%).

#### Model description

Median compliance with the model description domain was 100% (IQR 80–100%), the highest of the four domains. The majority of studies provided details about the model used (95%), initialization of model parameters (92%), training approach (78%) and method of selecting the final model (92%). The software libraries, frameworks and packages used were reported in 74%.

#### Model performance

Median compliance with the performance description domain was 60% (IQR 50–70%). A minority of studies reported testing on external data (22%) Almost all studies provided metrics of model performance (99.5%). Most studies provided statistical measures of significance and uncertainty when reporting results (78%). Many studies provided forms of robustness or sensitivity analysis (61%) and methods for explainability and interpretability (64%). A minority of studies reported failure analysis for incorrectly classified cases (32%). Most studies discussed their limitations (76%) and implications for practice (76%).

## Discussion

Poor reporting is a major source of research waste (10, 11) and ultimately may hinder advancement of AI research in the medical field. This systematic review evaluated the quality of reporting in AI studies involving automatic segmentation of structures on cardiac MRI. 209 studies were included from 2012 to early 2022. Each study was assessed for compliance with CLAIM, a checklist that attempts to provide a “best practice” framework for the reporting and publication of AI research in medical imaging (5). We identified major gaps in reporting and make a number of recommendations in order for this to be addressed (Table 1).

**TABLE 1:** Recommendations for study reporting. Main recommendations for AI study reporting are based on the gaps in the literature identified in this systematic review.

	Recommendation	Importance
*General*	Utilize a reporting framework (e.g., CLAIM).	Comparability of studies.
	Use of consistent and descriptive terminology.	Accessibility and comparability of studies.
*Data sources*	Describe the source of data, including patients’ eligibility criteria, their numbers and demographic and clinical characteristics.	Contextualizing model performance and generalizability.
	Clarify the number of scans and the flow of both patients and scans into different datasets (e.g., training, validation, and testing).	Understanding model performance and generalizability.
	Use publicly available datasets.	Comparability of models against a common benchmark.
*Model training and evaluation*	Describe the neural network, software packages and libraries in sufficient detail.	Study reproducibility.
	Define how the reference contours were generated, the experience of the annotator and annotation tools used.	Understanding model performance and generalizability.
	Explain the method of model training and performance.	Understanding model performance and generalizability.
	Test the model performance on external data with different characteristics to the training data.	Study and model reliability. Understanding model generalizability. Implementation in clinical practice.
	Perform failure analysis and report the limitations of the model.	Understanding model performance and generalizability.
	Publication of open-source code.	Understanding model performance and generalizability.

Accurate and sufficiently detailed descriptions of study materials and methods are of particular importance for AI studies in medical imaging to allow the assessment of reproducibility and reliability of results. Overall compliance with CLAIM was highest for the model description domain, with most studies providing a description of the model and details of training approaches. However, this was lowest for the dataset description domain, which indicated variable reporting of the data sources used to train and evaluate models.

A good understanding of data sources is a prerequisite for evaluating the validity of AI models. Although most studies identified their data sources, this was a significant omission in the studies that did not and one which greatly limits their interpretability. Public datasets were used in almost half of the studies, with the majority of these made available through segmentation challenges hosted by MICCAI (Supplementary Table 3). Public datasets contain previously de-identified and expertly contoured images, making them attractive to researchers. The proportion of studies using datasets from MICCAI challenges underlines its role as a driver for advancing the field. Importantly, the use of public datasets facilitates reproducibility and aids comparison between segmentation methods. However, public sources are not without their limitations. Public datasets consist of entirely retrospective data, which may place constraints on study design and model training. They are often small in size with limited demographic and clinical diversity, and therefore have inherent selection bias. Systematic biases affecting patient demographics are of serious concern in the application of AI methods to clinical practice. For example, a previous analysis of AI-based segmentation in CMR using a large-scale database found systematic bias for both sex and race (12) and similar biases have been reported for AI in radiographic imaging (13). The use of diverse datasets when training, validating and testing models is essential for generalizability and translation to clinical practice. A model trained on a dataset from one population does not guarantee equal performance on another. Multiple data sets, such as both retrospective and prospective, could be used in combination to improve the generalizability of AI models being trained. Even accounting for the use of public datasets, we found that few studies reported the intended sample size (which influences statistical power and reliability of results) or the demographic and clinical characteristics of the cases in each partition, (which indicates selection bias, confounders and generalizability). Providing summary information about the age and sex of cases is important, but may be insufficient in isolation. We noted that studies often lacked details about the proportions of cases with different pathologies, and the demographics for these groups. Furthermore, studies should not assume that readers are familiar with public datasets, and if these are used then detailed demographics and clinical characteristics should still be reported. The performance and validity of any model depend on the data on which it is trained and the data sources, including the rationale behind their choice and the intended sample size, should be clearly indicated. Study methodology must be reported in sufficient detail to enable accurate reproduction of results. Notably, the definition of the ground truth reference standard, the source of ground truth annotations and the annotation tools used were absent in a substantial number of studies. Understanding the structures included in the ground truth contours and the expertise of the annotator is essential in evaluating the training process and ultimately contextualizing the model’s performance. The proportions of studies that provided sufficiently detailed descriptions of the ground truth and its source were lower than expected for the field. For example, judging from the figures present in the included studies, ventricular trabeculations were usually included in the blood pool contours, although few studies described this process. Similarly, many studies failed to report the specific type of image used for ground truth annotation and model training and testing. While this could be inferred from figures, it remains essential information for understanding models and their generalizability. Finally, only a handful of studies indicated how missing data were handled and no studies indicated where a full study protocol could be accessed.

Detailed description of model training and performance is expected in this field. Testing model performance on external data was performed in less than a quarter of all studies. Model generalizability can only be fully evaluated when performance is assessed in demographic and clinical populations different from the original training cohort. The reported external datasets were small and captured only limited variations in imaging appearances. This represents a major hurdle to overcome before AI models can be implemented in clinical practice. We also noted subjectively that many publications used the terms “validation” and “test” interchangeably, or failed to distinguish these methods clearly. Regarding the use of data in AI studies, a validation set is used to optimize hyperparameters and performance between training epochs, while a testing set is used to assess the performance of the final model. The lack of consistent terminology in studies can limit the interpretability of their models and blur the distinction between internal holdout and external testing methods. Additionally, few studies reported failure analysis of incorrectly classified cases, suggesting that most did not explore the reasons for model underperformance. Furthermore, the vast majority of studies did not discuss the limitations of their methods, limiting their transparency. Open publishing of source code is a contentious topic in AI research and was only provided in one in ten of all studies. The public availability of code aids transparency, assists peer review and facilitates the development of new models, but bears important implications for ownership and rights.

The use of reporting frameworks, such as CLAIM, can be beneficial. For example, they may help to inform study design and highlight areas that may require rectification prior to dissemination of results. Frameworks assist standardization in reporting, improving comparability and interpretability by the wider scientific community. Study accessibility is also an important consideration in advancing the field. Regardless of journal type, AI studies in medical imaging need to cater for a broad potential readership, from clinicians to computer scientists. More standardized reporting and the use of consistent and accessible terminology are important in this regard.

We acknowledge limitations in this systematic review. Firstly, this review focused solely on AI segmentation in CMR studies. However, these findings are likely to apply to AI studies in other cardiac imaging modalities, such as echocardiogram, CT coronary angiography or nuclear myocardial perfusion studies. Furthermore, given that AI studies in chest imaging have shown similar shortcomings in reporting quality (14), our findings may be more broadly relevant to AI studies in medical imaging. Secondly, while our systematic search aimed to identify all published AI CMR segmentation studies, the body of unpublished, pre-print or technical conference literature is vast. A Github or arxiv.org search reveals numerous segmentation attempts of varying levels of reporting quality and beyond the scope of this review to capture. Thirdly, even despite the use of structured tools such as CLAIM, there remains an element of subjectivity in determining report quality, such as the amount of information required for a study to be deemed reproducible.

## Conclusion

This systematic review highlights the variability in reporting and identifies gaps in the existing literature of studies using AI segmentation of CMR images. We identified several key items that are missing in publications—most strikingly poor description of patients included in the training and validation of AI models and inadequate model failure analysis—which may limit study transparency, reproducibility and validity. This review supports closer adherence to established frameworks for reporting standards, such as CLAIM. In light of these findings, we have presented a number of recommendations for improving the quality of reporting of AI studies in both CMR and the wider field of cardiac imaging.

## Data availability statement

The original contributions presented in this study are included in the article/Supplementary Material, further inquiries can be directed to the corresponding author.

## Author contributions

SA and AS conceived the idea and need for the systematic review and contributed to the study conception and design. SD and SA performed the protocol registration in PROSPERO. SA created the search strategy and performed the literature search. SA, SD, MaS, MiS, and AqM performed screening and eligibility assessments independently. SA, SD, AhM, AqM, MaS, MiS, SJ, MG, VR, and HA evaluated the included studies and collected relevant data. SA, AhM, MaS, AqM, and SJ performed the material preparation and analysis. SA, AhM, SJ, and MaS drafted the manuscript, figures, and tables. SA, AhM, AqM, VR, and SD wrote the first draft of the manuscript. SA and AhM wrote the final draft, taking into account comments and suggestions from experts in the field AS, DO’R, HL, RG, MM, and PG. All authors contributed to the interpretation of data, commented on previous versions of the manuscript, read and approved the final manuscript, took part in the critical review and drafting of the manuscript, and have read and approved the final manuscript.

## Conflict of interest

The authors declare that the research was conducted in the absence of any commercial or financial relationships that could be construed as a potential conflict of interest.

## Publisher’s note

All claims expressed in this article are solely those of the authors and do not necessarily represent those of their affiliated organizations, or those of the publisher, the editors and the reviewers. Any product that may be evaluated in this article, or claim that may be made by its manufacturer, is not guaranteed or endorsed by the publisher.

## Abbreviations

AI: artificial intelligence
CLAIM: checklist for artificial intelligence in medical imaging
CMR: cardiac MRI
CT: computer tomography
DSC: dice similarity coefficient
PRISMA: preferred reporting items for systematic reviews and meta-analyses
PROSPERO: international prospective register of systematic reviews
MICCAI: medical image computing and computer-assisted intervention.

## References

[1] O’Regan DP. Putting machine learning into motion: applications in cardiovascular imaging. *Clin Radiol.* (2020) 75:33–7. 10.1016/j.crad.2019.04.008 31079952

[2] Reardon S. Rise of robot radiologists. *Nature.* (2019) 576:S54–8. 10.1038/d41586-019-03847-z 31853073

[3] Chen C Qin C Qiu H Tarroni G Duan J Bai W Deep learning for cardiac image segmentation: a review. *Front Cardiovasc Med.* (2020) 7:25. 10.3389/fcvm.2020.00025 32195270 PMC7066212

[4] Hosny A Parmar C Quackenbush J Schwartz LH Aerts HJWL. Artificial intelligence in radiology. *Nat Rev Cancer.* (2018) 18:500–10. 10.1038/s41568-018-0016-5 29777175 PMC6268174

[5] Mongan J Moy L Kahn CE Jr. Checklist for artificial intelligence in medical imaging (CLAIM): a guide for authors and reviewers. *Radiol Artif Intell.* (2020) 2:e200029. 10.1148/ryai.2020200029 33937821 PMC8017414

[6] Page MJ McKenzie JE Bossuyt PM Boutron I Hoffmann TC Mulrow CD The PRISMA 2020 statement: an updated guideline for reporting systematic reviews. *BMJ.* (2021) 372:n71. 10.1136/bmj.n71 33782057 PMC8005924

[7] Alabed S Alandejani F Dwivedi K Karunasaagarar K Sharkey M Garg P Validation of Artificial Intelligence Cardiac MRI Measurements: relationship to Heart Catheterization and Mortality Prediction. *Radiology.* (2022):212929. [Online ahead of print]. 10.1148/radiol.212929 35994400 PMC9523681

[8] Alandejani F Alabed S Garg P Goh ZM Karunasaagarar K Sharkey M Training and clinical testing of artificial intelligence derived right atrial cardiovascular magnetic resonance measurements. *J Cardiovasc Magnetic Resonan.* (2022) 24:25. 10.1186/s12968-022-00855-3 35387651 PMC8988415

[9] Fonseca CG Backhaus M Bluemke DA Britten RD Chung JD Cowan BR The Cardiac Atlas Project—an imaging database for computational modeling and statistical atlases of the heart. *Bioinformatics.* (2011) 27:2288–95. 10.1093/bioinformatics/btr360 21737439 PMC3150036

[10] Chalmers I Glasziou P. Avoidable waste in the production and reporting of research evidence. *Obstet Gynecol.* (2009) 114:1341–5. 10.1097/AOG.0b013e3181c3020d 19935040

[11] Glasziou P Chalmers I. Research waste is still a scandal—an essay by Paul Glasziou and Iain Chalmers. *BMJ.* (2018) 363:k4645. 10.1136/bmj.k4645

[12] Puyol-Antón E Ruijsink B Mariscal Harana J Piechnik SK Neubauer S Petersen SE Fairness in cardiac magnetic resonance imaging: assessing sex and racial bias in deep learning-based segmentation. *Front Cardiovasc Med*. (2022) 9:859310. 10.3389/fcvm.2022.859310 35463778 PMC9021445

[13] Larrazabal AJ Nieto N Peterson V Milone DH Ferrante E. Gender imbalance in medical imaging datasets produces biased classifiers for computer-aided diagnosis. *Proc Natl Acad Sci USA*. (2020) 117:12592–94. 10.1073/pnas.1919012117 32457147 PMC7293650

[14] Roberts M Driggs D Thorpe M Gilbey J Yeung M Ursprung S Common pitfalls and recommendations for using machine learning to detect and prognosticate for COVID-19 using chest radiographs and CT scans. *Nat Machine Intellig.* (2021) 3:199–217. 10.1038/s42256-021-00307-0

