# Supplementary material for: Quality of reporting in AI cardiac MRI segmentation studies – A systematic review and recommendations for future studies
Source: Front Cardiovasc Med. 2022 Jul 15;9:956811. doi: 10.3389/fcvm.2022.956811 (PMC9334661; doi:10.3389/fcvm.2022.956811)
Supplement: Supplementary file 1 [file Data_Sheet_1.docx]

# Supplementary Materials

## Database search strategy

Ovid (MEDLINE and EMBASE) Search Strategy:

1 automat*

2 exp ARTIFICIAL INTELLIGENCE/

3 exp MACHINE LEARNING/

4 exp DEEP LEARNING/

5 exp DEEP NEURAL NETWORK/

6 exp CONVOLUTION ALGORITHM/

7 ((deep or supervised or unsupervised or machine) and learning)

8 1 or 2 or 3 or 4 or 5 or 6 or 7

9 exp MAGNETIC RESONANCE IMAGING/

10 exp CARDIOVASCULAR MAGNETIC RESONANCE/

11 (Cine or MRI or MRA or (Magnetic and resonance))

12 9 or 10 or 11

13 (heart or cardi* or myocard* or coronar* or ventric* or LV or RV or atri*)

14 (segment* or conto* or annotat* or label*)

15 exp IMAGE SEGMENTATION/

16 exp SEGMENTATION ALGORITHM/

17 14 or 15 or 16

18 8 and 12 and 13 and 17

### **Supplementary Table 1**: Descriptive information about included studies

|  |  |  |  |  |  |  |  |  |  |  | **Compliance with CLAIM** | | | | |
| --- | --- | --- | --- | --- | --- | --- | --- | --- | --- | --- | --- | --- | --- | --- | --- |
| **Author** | **Year** | **Journal** | **Country** | **Images** | **Segmentation** | **Cases** | **Dataset** | **Validation** | **Performance evaluation** | **Public code** | **Study description domain** | **Dataset description domain** | **Model description domain** | **Performance domain** | **All domains** |
| Abdelaziz [(1)](https://paperpile.com/c/T2eW7l/m9koC) | 2015 | Technical | Algeria | Short axis | RV | 60 | Single centre | Internal holdout | Other metrics | No | 71% | 67% | 80% | 80% | 74% |
| Abdelrauof [(2)](https://paperpile.com/c/T2eW7l/qPLOM) | 2021 | Technical | Egypt | Multiple | Both ventricles | 250 | Multiple public | Internal holdout | DSC & other metrics | No | 63% | 44% | 100% | 70% | 62% |
| Abdeltawab [(3)](https://paperpile.com/c/T2eW7l/EAj2v) | 2020 | Technical | USA | Short axis | LV | 176 | Public & single centre | External testing | DSC & other metrics | No | 50% | 50% | 80% | 50% | 54% |
| Afshin [(4)](https://paperpile.com/c/T2eW7l/4wm9i) | 2014 | Technical | Canada | Short axis | LV | 58 | Single centre | Internal holdout | Other metrics | No | 71% | 64% | 100% | 70% | 73% |
| Alandejani [(5)](https://paperpile.com/c/T2eW7l/eINfB) | 2022 | Medical | UK | Four chamber | RA | 365 | Multi-centre | Internal holdout | DSC | No | 71% | 73% | 80% | 70% | 73% |
| Albà [(6)](https://paperpile.com/c/T2eW7l/owg5Z) | 2015 | Technical | Spain | Short axis | Both ventricles | 60 | Single centre | Internal holdout | Other metrics | No | 75% | 63% | 100% | 100% | 79% |
| Albà [(7)](https://paperpile.com/c/T2eW7l/L0ad2) | 2018 | Technical | Spain | Short axis | LV | 1200 | Public | Internal holdout | Other metrics | No | 71% | 64% | 80% | 40% | 61% |
| Ammar [(8)](https://paperpile.com/c/T2eW7l/otstX) | 2021 | Technical | Morocco | Short axis | Both ventricles & myocardium | 150 | Public | Internal holdout | DSC & other metrics | No | 86% | 64% | 80% | 50% | 67% |
| Ammar [(9)](https://paperpile.com/c/T2eW7l/EV2uj) | 2012 | Technical | Algeria | Short axis | LV | 45 | Public | Internal holdout | Other metrics | No | 86% | 64% | 100% | 60% | 73% |
| Ankenbrand [(10)](https://paperpile.com/c/T2eW7l/JHS9D) | 2021 | Medical | Germany | Short axis | LV | 1140 | Public | Internal holdout | DSC | No | 86% | 64% | 100% | 40% | 67% |
| Atehortúa [(11)](https://paperpile.com/c/T2eW7l/iVFEC) | 2016 | Technical | Colombia | Short axis | RV | 48 | Public & multicentre | Internal holdout | DSC & other metrics | No | 75% | 75% | 100% | 50% | 72% |
| Attar [(12)](https://paperpile.com/c/T2eW7l/6dyDM) | 2019 | Technical | UK | Multiple | Both ventricles | 4275 | Public | Internal holdout | DSC & other metrics | No | 86% | 55% | 80% | 60% | 67% |
| Augusto [(13)](https://paperpile.com/c/T2eW7l/iHjgh) | 2021 | Hybrid | UK | Short axis | Myocardium | 1983 | Multicentre | External testing | Other metrics | No | 71% | 64% | 100% | 60% | 70% |
| Avendi [(14)](https://paperpile.com/c/T2eW7l/3oHVF) | 2016 | Technical | USA | Short axis | LV | 45 | Public | Internal holdout | DSC & other metrics | No | 88% | 75% | 40% | 90% | 77% |
| Avendi [(15)](https://paperpile.com/c/T2eW7l/lanz9) | 2017 | Medical | USA | Short axis | RV | 32 | Public | Internal holdout | DSC & other metrics | No | 86% | 64% | 100% | 70% | 76% |
| Bai [(16)](https://paperpile.com/c/T2eW7l/OKlUp) | 2018 | Medical | UK | Multiple | Four chambers | 4875 | Public | Internal holdout | DSC & other metrics | Yes | 86% | 58% | 100% | 70% | 74% |
| Barba-J [(17)](https://paperpile.com/c/T2eW7l/ZZvRU) | 2018 | Hybrid | Mexico | Short axis | LV | 15 | Public | External testing | DSC & other metrics | No | 75% | 81% | 100% | 90% | 85% |
| Bartoli [(18)](https://paperpile.com/c/T2eW7l/yAJi3) | 2020 | Hybrid | France | Short axis | LV | 449 | Multicentre | External testing | DSC & other metrics | No | 75% | 44% | 0% | 40% | 44% |
| Beache [(19)](https://paperpile.com/c/T2eW7l/vc9Hm) | 2014 | Technical | USA | Short axis | LV | 8 | NR | Internal holdout | DSC | No | 71% | 67% | 100% | 80% | 76% |
| Bhatt [(20)](https://paperpile.com/c/T2eW7l/XlCtH) | 2021 | Technical | Canada | Short axis | Myocardium | 11 | NR | Internal holdout | DSC | No | 71% | 73% | 80% | 60% | 70% |
| Bhuva [(21)](https://paperpile.com/c/T2eW7l/axJMK) | 2019 | Medical | UK | Short axis | LV | 709 | Multicentre | External testing | Other metrics | No | 38% | 56% | 80% | 60% | 56% |
| Borra [(22)](https://paperpile.com/c/T2eW7l/zJQYB) | 2020 | Medical | Italy | Axial | LA | 100 | Public | Internal holdout | DSC & other metrics | No | 63% | 44% | 100% | 70% | 62% |
| Brahim [(23)](https://paperpile.com/c/T2eW7l/1jZpl) | 2021 | Technical | Tunisia | Short axis | Myocardium & scar | 150 | Public | Internal holdout | DSC & other metrics | Yes | 50% | 44% | 80% | 50% | 51% |
| Brodoefel [(24)](https://paperpile.com/c/T2eW7l/9KfTq) | 2012 | Medical | Germany | Short axis | LV | 20 | Single centre | Internal holdout | Other metrics | No | 75% | 56% | 100% | 60% | 67% |
| Budai [(25)](https://paperpile.com/c/T2eW7l/7Vsao) | 2020 | Technical | Hungary | Short axis | Both ventricles | 5570 | Public & single centre | External testing | DSC & other metrics | Yes | 75% | 44% | 80% | 60% | 59% |
| Campello [(26)](https://paperpile.com/c/T2eW7l/6KR9T) | 2021 | Technical | Spain | Short axis | Both ventricles | 375 | Multicentre | Internal holdout | DSC & other metrics | No | 88% | 50% | 60% | 50% | 59% |
| Carbajal-Degante [(27)](https://paperpile.com/c/T2eW7l/vOKUi) | 2021 | Technical | Mexico | NR | Both ventricles | NR | Public | Internal holdout | DSC & other metrics | No | 71% | 64% | 100% | 70% | 73% |
| Cardenas [(28)](https://paperpile.com/c/T2eW7l/Z2t0w) | 2020 | Technical | Argentina | Short axis | Myocardium | 15 | Public | Internal holdout | DSC | No | 50% | 38% | 80% | 40% | 46% |
| Carminati [(29)](https://paperpile.com/c/T2eW7l/AYQxe) | 2016 | Medical | Italy | Short axis | Scar | 20 | Single centre | Internal holdout | DSC | No | 75% | 44% | 100% | 90% | 70% |
| Chang [(30)](https://paperpile.com/c/T2eW7l/j7xCb) | 2020 | Technical | China | Short axis | Both ventricles | 150 | Public | Internal holdout | DSC & other metrics | No | 71% | 64% | 80% | 40% | 61% |
| Chen [(31)](https://paperpile.com/c/T2eW7l/LnM7y) | 2021 | Medical | USA | Short axis | LV | 5143 | Public & single centre | External testing | DSC | Yes | 50% | 56% | 60% | 50% | 54% |
| Chen [(32)](https://paperpile.com/c/T2eW7l/TNoPT) | 2018 | Technical | China | NR | RV | 145 | Single centre | Internal holdout | DSC | No | 57% | 64% | 60% | 20% | 48% |
| Chenoune [(33)](https://paperpile.com/c/T2eW7l/atS9s) | 2012 | Technical | France | Short axis | Myocardium | 10 | Single centre | Internal holdout | Other metrics | No | 63% | 63% | 100% | 90% | 74% |
| Cui [(34)](https://paperpile.com/c/T2eW7l/cttKC) | 2021 | Technical | China | Short axis | LV | 200 | Public | Internal holdout | DSC & other metrics | No | 63% | 69% | 100% | 80% | 74% |
| Curiale [(35)](https://paperpile.com/c/T2eW7l/igrt5) | 2019 | Technical | Argentina | Short axis | LV | 140 | Public | Internal holdout | DSC & other metrics | No | 57% | 67% | 80% | 80% | 71% |
| Curialea [(35)](https://paperpile.com/c/T2eW7l/igrt5) | 2019 | Technical | Argentina | Short axis | LV | 140 | Multiple public | Internal holdout | DSC & other metrics | No | 88% | 38% | 100% | 50% | 59% |
| Davies [(36)](https://paperpile.com/c/T2eW7l/o5fkD) | 2022 | Medical | UK | Multiple | LV & myocardium | 3,309 | Multicentre | External testing | Other metrics | No | 63% | 56% | 80% | 80% | 67% |
| Daviller [(37)](https://paperpile.com/c/T2eW7l/iOvJT) | 2019 | Technical | France | Short axis | Myocardium | 30 | Single centre | Internal holdout | DSC & other metrics | No | 71% | 67% | 100% | 70% | 74% |
| Dharanibai [(38)](https://paperpile.com/c/T2eW7l/jZAO7) | 2014 | Technical | India | Short axis | LV | 30 | Public | Internal holdout | DSC & other metrics | No | 71% | 45% | 83% | 60% | 62% |
| Diller [(39)](https://paperpile.com/c/T2eW7l/C1xu2) | 2020 | Hybrid | Germany | Multiple | Both ventricles | 395 | Multicentre | External testing | DSC & other metrics | No | 75% | 38% | 100% | 20% | 49% |
| Do [(40)](https://paperpile.com/c/T2eW7l/Uf8hb) | 2020 | Medical | USA | Short axis | Myocardium | 22 | Single centre | Internal holdout | DSC & other metrics | No | 63% | 25% | 60% | 40% | 41% |
| Dreijer [(41)](https://paperpile.com/c/T2eW7l/hxJIt) | 2013 | Hybrid | South Africa | Short axis | LV | 78 | Multiple public | External testing | DSC & other metrics | No | 63% | 44% | 100% | 50% | 56% |
| Du [(42)](https://paperpile.com/c/T2eW7l/N1NXv) | 2020 | Technical | China | Short axis | Myocardium | 20 | Public | External testing | DSC & other metrics | No | 63% | 56% | 100% | 50% | 62% |
| Du [(43)](https://paperpile.com/c/T2eW7l/e9i8c) | 2021 | Technical | China | Two chamber | RV | 68 | Public | Internal holdout | DSC & other metrics | No | 86% | 75% | 100% | 70% | 79% |
| Du [(44)](https://paperpile.com/c/T2eW7l/Y5e9Y) | 2019 | Hybrid | China | Short axis | LV | 145 | Multicentre | Internal holdout | DSC | No | 86% | 67% | 100% | 80% | 79% |
| Du [(45)](https://paperpile.com/c/T2eW7l/sLMS6) | 2020 | Hybrid | China | NR | LA | 100 | Public | Internal holdout | DSC & other metrics | No | 71% | 64% | 100% | 50% | 67% |
| El-Rewaidy [(46)](https://paperpile.com/c/T2eW7l/zrCgE) | 2022 | Medical | USA | Short axis | RV | 935 | Public & single centre | External testing | DSC & other metrics | No | 88% | 75% | 100% | 90% | 85% |
| Eslami [(47)](https://paperpile.com/c/T2eW7l/Vvbxb) | 2013 | Technical | Germany | Short axis | LV | 104 | Single centre | Internal holdout | DSC & other metrics | No | 88% | 50% | 100% | 60% | 67% |
| Fadil [(48)](https://paperpile.com/c/T2eW7l/oMDAU) | 2021 | Medical | Singapore | Short axis | Both ventricles | 619 | NR | External testing | DSC & other metrics | No | 88% | 69% | 100% | 90% | 82% |
| Fahmy [(49)](https://paperpile.com/c/T2eW7l/lZTJ3) | 2019 | Medical | USA | Short axis | Myocardium | 665 | Single centre | Internal holdout | DSC & other metrics | No | 71% | 64% | 100% | 40% | 64% |
| Fahmy [(50)](https://paperpile.com/c/T2eW7l/bNklm) | 2021 | Medical | USA | Short axis | Scar | 191 | Multicentre | External testing | Other metrics | No | 57% | 64% | 20% | 50% | 52% |
| Fahmy [(51)](https://paperpile.com/c/T2eW7l/IVrsN) | 2020 | Medical | USA | Short axis | Scar | 1073 | Multicentre | Internal holdout | DSC & other metrics | No | 75% | 56% | 20% | 90% | 64% |
| Farrag [(52)](https://paperpile.com/c/T2eW7l/3AN4H) | 2021 | Technical | Canada | Multiple | Myocardium | 60 | Single centre | Internal holdout | DSC | No | 71% | 64% | 100% | 70% | 73% |
| Feng [(53)](https://paperpile.com/c/T2eW7l/4uNRv) | 2018 | Technical | Singapore | Multiple | Myocardium | 30 | Single centre | Internal holdout | Other metrics | No | 75% | 75% | 100% | 80% | 79% |
| Feng [(54)](https://paperpile.com/c/T2eW7l/Beai5) | 2016 | Technical | China | Short axis | LV | 45 | Public | External testing | DSC | No | 86% | 64% | 100% | 80% | 79% |
| Ferreira [(55)](https://paperpile.com/c/T2eW7l/pd1LO) | 2020 | Medical | UK | Short axis | LV | 492 | NR | Internal holdout | DSC | No | 71% | 73% | 80% | 40% | 64% |
| Freling [(56)](https://paperpile.com/c/T2eW7l/SJkaA) | 2013 | Medical | Netherl&s | Multiple | RV | 65 | Single centre | Internal holdout | Other metrics | No | 63% | 63% | 100% | 80% | 72% |
| Gao [(57)](https://paperpile.com/c/T2eW7l/1ZHEQ) | 2013 | Medical | UK | Short axis | LV | 25 | Single centre | Internal holdout | DSC | No | 86% | 67% | 100% | 80% | 79% |
| Ghadimi [(58)](https://paperpile.com/c/T2eW7l/PPc84) | 2021 | Medical | USA | Short axis | Both ventricles | 108 | Multicentre | Internal holdout | DSC | Yes | 86% | 67% | 100% | 80% | 79% |
| Giannakidis [(59)](https://paperpile.com/c/T2eW7l/PNYMS) | 2015 | Technical | UK | Short axis | Scar | 13 | Single centre | Internal holdout | Other metrics | No | 88% | 81% | 80% | 80% | 82% |
| Gonzales [(60)](https://paperpile.com/c/T2eW7l/oi1ev) | 2021 | Hybrid | USA | Multiple | LA | 37 | Single centre | Internal holdout | DSC & other metrics | No | 88% | 63% | 100% | 80% | 77% |
| Goyal [(61)](https://paperpile.com/c/T2eW7l/zvYON) | 2020 | Medical | USA | Short axis | LV | 21 | Single centre | Internal holdout | Other metrics | No | 57% | 73% | 80% | 70% | 70% |
| Goyal [(62)](https://paperpile.com/c/T2eW7l/a3bDT) | 2019 | Hybrid | USA | NR | Both ventricles | NR | Single centre | Internal holdout | DSC | No | 86% | 67% | 100% | 90% | 82% |
| Guo [(63)](https://paperpile.com/c/T2eW7l/9z5pN) | 2020 | Technical | Canada | Short axis | Both ventricles & myocardium | NR | Public | Internal holdout | DSC | No | 71% | 64% | 100% | 60% | 70% |
| Gupta [(64)](https://paperpile.com/c/T2eW7l/PnAZ3) | 2018 | Medical | Sweden | Short axis | LV | 20 | NR | Internal holdout | DSC & other metrics | No | 71% | 64% | 100% | 20% | 58% |
| Hajiaghayi [(65)](https://paperpile.com/c/T2eW7l/KAXmC) | 2017 | Technical | USA | Short axis | LV | 33 | Single centre | Internal holdout | DSC & other metrics | No | 86% | 67% | 100% | 60% | 74% |
| Hann [(66)](https://paperpile.com/c/T2eW7l/Vl6du) | 2021 | Technical | UK | Short axis | LV | NR | Public & single centre | External testing | DSC | No | 86% | 67% | 100% | 70% | 76% |
| Hautvast [(67)](https://paperpile.com/c/T2eW7l/6PAWG) | 2012 | Medical | Netherl&s | Short axis | LV | 1555 | Public | Internal holdout | Other metrics | No | 71% | 67% | 100% | 70% | 74% |
| He [(68)](https://paperpile.com/c/T2eW7l/9cJ1R) | 2020 | Technical | China | Short axis | LV | 100 | Public | Internal holdout | DSC & other metrics | No | 86% | 67% | 100% | 70% | 76% |
| Heidenreich [(69)](https://paperpile.com/c/T2eW7l/6RvOW) | 2021 | Medical | Germany | Short axis | Scar | 75 | Single centre | Internal holdout | DSC & other metrics | No | 63% | 56% | 100% | 70% | 67% |
| Ho [(70)](https://paperpile.com/c/T2eW7l/HM0JA) | 2021 | Medical | Korea | Short axis | LV | 300 | Public | Internal holdout | Other metrics | No | 88% | 69% | 100% | 80% | 79% |
| Hu [(71)](https://paperpile.com/c/T2eW7l/3FvUJ) | 2019 | Technical | China | Short axis | LV | 900 | Public & single centre | External testing | DSC | No | 86% | 67% | 100% | 40% | 68% |
| Hu [(72)](https://paperpile.com/c/T2eW7l/Cdb8G) | 2014 | Medical | China | Short axis | LV | 45 | Public | Internal holdout | DSC & other metrics | No | 88% | 63% | 100% | 70% | 74% |
| Hu [(73)](https://paperpile.com/c/T2eW7l/e7OYd) | 2013 | Medical | China | Short axis | LV | 45 | Public | Internal holdout | DSC | No | 88% | 50% | 40% | 80% | 64% |
| Huang [(74)](https://paperpile.com/c/T2eW7l/O6cH7) | 2017 | Medical | Taiwan | Short axis | LV | 10 | Single centre | Internal holdout | Other metrics | No | 75% | 56% | 100% | 60% | 67% |
| Isensee [(75)](https://paperpile.com/c/T2eW7l/KYCwI) | 2018 | Technical | Germany | Short axis | Both ventricles & myocardium | 150 | Public | Internal holdout | DSC | No | 71% | 64% | 100% | 50% | 67% |
| Kadir [(76)](https://paperpile.com/c/T2eW7l/2Xoxu) | 2012 | Technical | UK | Short axis | LV | 12 | Single centre | Internal holdout | Other metrics | No | 88% | 69% | 100% | 70% | 77% |
| Kar [(77)](https://paperpile.com/c/T2eW7l/o2GOK) | 2021 | Medical | USA | Short axis | LV | 42 | Single centre | Internal holdout | DSC & other metrics | Yes | 57% | 45% | 80% | 70% | 61% |
| Kar [(78)](https://paperpile.com/c/T2eW7l/QEuBE) | 2021 | Medical | USA | Short axis | LV | 42 | Single centre | Internal holdout | DSC & other metrics | Yes | 63% | 50% | 100% | 50% | 59% |
| Kar [(79)](https://paperpile.com/c/T2eW7l/SGAr9) | 2020 | Technical | USA | Short axis | LV | 42 | Single centre | N/A | Other metrics | Yes | 88% | 50% | 80% | 50% | 62% |
| Khalifa [(80)](https://paperpile.com/c/T2eW7l/rLvWj) | 2012 | Technical | USA | NR | LV | 26 | Single centre | External testing | DSC & other metrics | No | 71% | 75% | 80% | 90% | 79% |
| Khamechian [(81)](https://paperpile.com/c/T2eW7l/cROpB) | 2018 | Medical | Iran | Short axis | Myocardium | 37 | Public & single centre | External testing | Other metrics | No | 88% | 56% | 100% | 90% | 77% |
| Khened [(82)](https://paperpile.com/c/T2eW7l/hgFAH) | 2019 | Technical | India | Short axis | Both ventricles & myocardium | 350 | Multiple public | External testing | DSC & other metrics | No | 57% | 64% | 80% | 40% | 58% |
| Kim [(83)](https://paperpile.com/c/T2eW7l/F5FMS) | 2020 | Technical | Republic of Korea | Short axis | Myocardium | 145 | Public & single centre | External testing | DSC | No | 75% | 44% | 100% | 90% | 69% |
| Kim [(84)](https://paperpile.com/c/T2eW7l/SIuC4) | 2019 | Technical | South Korea | Short axis | Myocardium | 110 | Public & single centre | Internal holdout | DSC | Yes | 88% | 44% | 100% | 90% | 72% |
| Koehler [(85)](https://paperpile.com/c/T2eW7l/M8iMi) | 2021 | Technical | Germany | Multiple | RV | 294 | Public & multicentre | Internal holdout | DSC | Yes | 75% | 44% | 100% | 50% | 59% |
| Kumar [(86)](https://paperpile.com/c/T2eW7l/Rukm6) | 2020 | Medical | India | NR | LV | NR | Single centre | Internal holdout | DSC & other metrics | No | 57% | 64% | 80% | 40% | 58% |
| Kurzendorfer [(87)](https://paperpile.com/c/T2eW7l/lGryz) | 2017 | Technical | Germany | Short axis | Scar | 30 | Multicentre | Internal holdout | DSC | No | 50% | 50% | 100% | 60% | 59% |
| Lebenberg [(88)](https://paperpile.com/c/T2eW7l/pKNRH) | 2012 | Technical | France | Short axis | LV | 45 | Public | Internal holdout | Other metrics | No | 63% | 63% | 80% | 40% | 59% |
| Li [(89)](https://paperpile.com/c/T2eW7l/Z64yY) | 2019 | Technical | China | NR | RV | 48 | NR | Internal holdout | DSC | No | 38% | 50% | 80% | 60% | 54% |
| Li [(90)](https://paperpile.com/c/T2eW7l/sxaaf) | 2020 | Technical | China | NR | Scar | 58 | Single centre | Internal holdout | DSC & other metrics | No | 38% | 56% | 80% | 60% | 56% |
| Liao [(91)](https://paperpile.com/c/T2eW7l/esyVm) | 2017 | Technical | China | Short axis | LV | 1140 | Multiple public | Internal holdout | Other metrics | No | 63% | 50% | 100% | 30% | 54% |
| Lin [(92)](https://paperpile.com/c/T2eW7l/OtGie) | 2020 | Medical | China | Short axis | LV | 45 | Public | Internal holdout | DSC & other metrics | No | 38% | 50% | 100% | 70% | 59% |
| Lindsey [(93)](https://paperpile.com/c/T2eW7l/VkqIF) | 2020 | Technical | USA | Short axis | Both ventricles & myocardium | 100 | Public | Internal holdout | DSC & other metrics | No | 63% | 56% | 80% | 50% | 59% |
| Liu [(94)](https://paperpile.com/c/T2eW7l/eK662) | 2019 | Technical | China | NR | Myocardium | 32 | Single centre | Internal holdout | DSC | No | 71% | 64% | 80% | 50% | 64% |
| Liu [(95)](https://paperpile.com/c/T2eW7l/2N6Ip) | 2020 | Technical | China | NR | Both ventricles | 85 | NR | Internal holdout | Other metrics | No | 63% | 63% | 80% | 60% | 64% |
| Liu [(96)](https://paperpile.com/c/T2eW7l/kR9oU) | 2012 | Medical | China | Short axis | LV | 45 | Single centre | Internal holdout | DSC | No | 50% | 38% | 60% | 50% | 46% |
| Liu [(97)](https://paperpile.com/c/T2eW7l/9RNai) | 2018 | Hybrid | China | Short axis | LV | 30 | Single centre | External testing | DSC | No | 71% | 64% | 100% | 60% | 70% |
| Liu [(98)](https://paperpile.com/c/T2eW7l/zIaVI) | 2017 | Technical | China | Short axis | Myocardium | 33 | Single centre | Internal holdout | DSC & other metrics | No | 50% | 81% | 100% | 70% | 74% |
| Luo [(99)](https://paperpile.com/c/T2eW7l/hUSqP) | 2020 | Medical | China | NR | Both ventricles & myocardium | 250 | Public & single centre | External testing | DSC & other metrics | No | 88% | 69% | 100% | 50% | 72% |
| Luo [(100)](https://paperpile.com/c/T2eW7l/pvVxo) | 2021 | Hybrid | China | NR | RV | 45 | Single centre | Internal holdout | DSC & other metrics | No | 75% | 50% | 80% | 50% | 59% |
| Luo [(101)](https://paperpile.com/c/T2eW7l/PmZ8L) | 2015 | Medical | China | Short axis | LV | 26 | Single centre | Internal holdout | Other metrics | No | 14% | 73% | 60% | 30% | 45% |
| Luo [(102)](https://paperpile.com/c/T2eW7l/6qyiM) | 2020 | Technical | China | Short axis | Both ventricles | 428 | Public | Internal holdout | Other metrics | No | 38% | 44% | 80% | 20% | 41% |
| Ma [(103)](https://paperpile.com/c/T2eW7l/R30M8) | 2019 | Technical | China | Short axis | Both ventricles & myocardium | 150 | Multiple public | External testing | DSC & other metrics | No | 43% | 64% | 80% | 40% | 55% |
| Ma [(104)](https://paperpile.com/c/T2eW7l/LIYqF) | 2017 | Hybrid | China | Multiple | LA | NR | Public | External testing | DSC & other metrics | No | 38% | 75% | 100% | 60% | 67% |
| Ma [(105)](https://paperpile.com/c/T2eW7l/p2Ghs) | 2016 | Hybrid | China | Short axis | LV | 45 | Public | Internal holdout | DSC | No | 63% | 69% | 100% | 70% | 72% |
| Ma [(106)](https://paperpile.com/c/T2eW7l/o06kk) | 2021 | Technical | China | Short axis | RV | 30 | Single centre | Internal holdout | DSC & other metrics | No | 50% | 44% | 80% | 60% | 54% |
| Mahapatra [(107)](https://paperpile.com/c/T2eW7l/JWPVr) | 2013 | Technical | Switzerl& | NR | LV | 30 | Public | Internal holdout | DSC & other metrics | No | 63% | 56% | 100% | 40% | 59% |
| Mahapatra [(108)](https://paperpile.com/c/T2eW7l/K20PW) | 2013 | Technical | Switzerl& | NR | Both ventricles | 30 | Public | Internal holdout | DSC & other metrics | No | 57% | 64% | 100% | 70% | 70% |
| Mahapatra [(109)](https://paperpile.com/c/T2eW7l/uM6at) | 2014 | Technical | Switzerl& | Short axis | RV | 32 | Single centre | Internal holdout | DSC | No | 50% | 63% | 100% | 70% | 67% |
| Mamalakis [(110)](https://paperpile.com/c/T2eW7l/Btr4y) | 2021 | Technical | UK | Short axis | Myocardium & scar | 60 | Multiple public | External testing | DSC | Yes | 71% | 73% | 80% | 70% | 73% |
| Matthew [(111)](https://paperpile.com/c/T2eW7l/JEmzD) | 2012 | Medical | UK | Short axis | LV | 15 | Single centre | Internal holdout | Other metrics | No | 71% | 73% | 100% | 80% | 79% |
| Moccia [(112)](https://paperpile.com/c/T2eW7l/t7Q51) | 2019 | Technical | Italy | Short axis | Scar | 30 | Single centre | Internal holdout | DSC | No | 43% | 64% | 100% | 60% | 64% |
| Morais [(113)](https://paperpile.com/c/T2eW7l/mCnFk) | 2017 | Technical | Portugal | Short axis | Myocardium | 75 | Multicentre | Internal holdout | DSC & other metrics | No | 43% | 73% | 100% | 70% | 70% |
| Morris [(114)](https://paperpile.com/c/T2eW7l/yCytR) | 2018 | Hybrid | USA | Four chamber | Four chambers | 31 | Single centre | Internal holdout | DSC & other metrics | No | 50% | 19% | 100% | 30% | 38% |
| Nambakhsh [(115)](https://paperpile.com/c/T2eW7l/5OEWh) | 2013 | Technical | Canada | Short axis | LV | 20 | Single centre | Internal holdout | DSC & other metrics | No | 63% | 56% | 100% | 80% | 69% |
| Ngo [(116)](https://paperpile.com/c/T2eW7l/uXsCl) | 2017 | Technical | Vietnam | Short axis | LV | 45 | Public | Internal holdout | DSC | No | 71% | 64% | 100% | 80% | 76% |
| Niu [(117)](https://paperpile.com/c/T2eW7l/8boTy) | 2019 | Technical | China | Short axis | Myocardium | 83 | Public | Internal holdout | Other metrics | No | 78% | 56% | 80% | 70% | 68% |
| Oktay [(118)](https://paperpile.com/c/T2eW7l/aZ10J) | 2017 | Technical | UK | Short axis | Both ventricles | 50 | Public | Internal holdout | DSC & other metrics | No | 63% | 44% | 100% | 80% | 64% |
| Paknezhad [(119)](https://paperpile.com/c/T2eW7l/INPQB) | 2016 | Technical | Singapore | Two chamber | LV | 51 | NR | Internal holdout | Other metrics | No | 63% | 44% | 100% | 80% | 64% |
| Penso [(120)](https://paperpile.com/c/T2eW7l/TnJbJ) | 2021 | Technical | Italy | Short axis | Both ventricles | 220 | Single centre | External testing | DSC & other metrics | No | 88% | 63% | 100% | 60% | 72% |
| Pérez-Pelegrí [(121)](https://paperpile.com/c/T2eW7l/rcmrB) | 2021 | Technical | Spain | Short axis | LV | 397 | Single centre | Internal holdout | DSC | No | 88% | 44% | 80% | 50% | 59% |
| Punithakumar [(122)](https://paperpile.com/c/T2eW7l/Heuqv) | 2013 | Technical | Canada | Short axis | LV | 58 | Single centre | Internal holdout | Other metrics | No | 88% | 56% | 80% | 60% | 67% |
| Punithakumar [(123)](https://paperpile.com/c/T2eW7l/eryhC) | 2015 | Technical | Canada | Short axis | RV | 48 | Public | External testing | Other metrics | No | 75% | 44% | 60% | 50% | 54% |
| Puyol-Anton [(124)](https://paperpile.com/c/T2eW7l/ZwlYb) | 2020 | Medical | UK | Short axis | LV | 900 | Public | Internal holdout | DSC | No | 50% | 44% | 100% | 60% | 56% |
| Qin [(125)](https://paperpile.com/c/T2eW7l/fxyry) | 2020 | Technical | China | Short axis | LV | 30 | Single centre | Internal holdout | DSC | No | 63% | 50% | 100% | 40% | 56% |
| Queiros [(126)](https://paperpile.com/c/T2eW7l/RR5BQ) | 2017 | Technical | Portugal | Short axis | LV | 45 | Public | Internal holdout | DSC & other metrics | No | 57% | 73% | 80% | 60% | 67% |
| Queirós [(127)](https://paperpile.com/c/T2eW7l/DFUL9) | 2014 | Technical | Belgium | Short axis | LV | 45 | Public | Internal holdout | DSC & other metrics | No | 63% | 50% | 80% | 60% | 59% |
| Queirós [(128)](https://paperpile.com/c/T2eW7l/DlDxy) | 2016 | Medical | Portugal | Short axis | Myocardium | 318 | Multicentre | Internal holdout | Other metrics | No | 71% | 64% | 80% | 70% | 70% |
| Razeghi [(129)](https://paperpile.com/c/T2eW7l/TfzxN) | 2020 | Medical | UK | Axial | LA | 396 | Public & single centre | External testing | DSC | Yes | 75% | 44% | 100% | 70% | 64% |
| Ringenberg [(130)](https://paperpile.com/c/T2eW7l/4cKM2) | 2014 | Technical | USA | Short axis | RV | 48 | Public | Internal holdout | DSC | No | 71% | 64% | 80% | 60% | 67% |
| Romaguera [(131)](https://paperpile.com/c/T2eW7l/fWzfS) | 2018 | Technical | Brazil | Short axis | Myocardium | 45 | Public | Internal holdout | DSC & other metrics | No | 63% | 44% | 80% | 50% | 54% |
| Rostami [(132)](https://paperpile.com/c/T2eW7l/LC2lA) | 2020 | Technical | Iran | NR | LV | 45 | Public | Internal holdout | DSC & other metrics | No | 50% | 38% | 80% | 60% | 51% |
| Ruijsink [(133)](https://paperpile.com/c/T2eW7l/fPGbw) | 2020 | Medical | UK | Multiple | Both ventricles | 2829 | Public & single centre | External testing | DSC & other metrics | Yes | 50% | 63% | 100% | 80% | 69% |
| Sander [(134)](https://paperpile.com/c/T2eW7l/0ohoj) | 2020 | Medical | Netherl&s | Short axis | Both ventricles & myocardium | 100 | Public | Internal holdout | DSC & other metrics | Yes | 71% | 64% | 80% | 50% | 64% |
| Sandfort [(135)](https://paperpile.com/c/T2eW7l/A7rRN) | 2021 | Medical | USA | NR | LV | 70 | Single centre | Internal holdout | DSC & other metrics | No | 63% | 56% | 60% | 40% | 54% |
| Scannell [(136)](https://paperpile.com/c/T2eW7l/54Yh2) | 2020 | Medical | UK | Short axis | Myocardium | 175 | Single centre | Internal holdout | DSC | No | 63% | 69% | 100% | 40% | 64% |
| Shaaf [(137)](https://paperpile.com/c/T2eW7l/7aM9k) | 2022 | Medical | Malaysia | Short axis | LV | 150 | Public | Internal holdout | DSC & other metrics | No | 63% | 38% | 80% | 50% | 51% |
| Shahzad [(138)](https://paperpile.com/c/T2eW7l/IY03e) | 2017 | Technical | Netherl&s | Four chamber | LV | 145 | Multicentre | Internal holdout | DSC | No | 38% | 44% | 80% | 60% | 51% |
| Sharma [(139)](https://paperpile.com/c/T2eW7l/UooZ0) | 2020 | Technical | Australia | NR | LV | 5051 | Public | Internal holdout | DSC & other metrics | No | 75% | 38% | 80% | 40% | 51% |
| Shen [(140)](https://paperpile.com/c/T2eW7l/P9tHq) | 2021 | Medical | USA | Short axis | Both ventricles | 99 | Single centre | Internal holdout | DSC & other metrics | No | 75% | 44% | 80% | 60% | 59% |
| Shi [(141)](https://paperpile.com/c/T2eW7l/jZvOm) | 2021 | Technical | China | Short axis | Both ventricles | NR | Public | External testing | DSC & other metrics | No | 57% | 64% | 100% | 40% | 61% |
| Shi [(142)](https://paperpile.com/c/T2eW7l/mT4cC) | 2021 | Medical | China | Short axis | Myocardium | 150 | Public | Internal holdout | DSC | No | 75% | 63% | 80% | 70% | 69% |
| Simantiris [(143)](https://paperpile.com/c/T2eW7l/AZJFL) | 2020 | Technical | Greece | NR | Both ventricles & myocardium | NR | Public | Internal holdout | DSC | No | 63% | 69% | 80% | 50% | 64% |
| Sliman [(144)](https://paperpile.com/c/T2eW7l/HUBV8) | 2013 | Technical | USA | Short axis | LV | 15 | Single centre | External testing | DSC & other metrics | No | 75% | 44% | 100% | 60% | 62% |
| Suinesiaputra [(145)](https://paperpile.com/c/T2eW7l/lAFfG) | 2015 | Technical | New Zeal& | Short axis | LV | 95 | Public | Internal holdout | DSC & other metrics | No | 71% | 64% | 80% | 60% | 67% |
| Suinesiaputra [(146)](https://paperpile.com/c/T2eW7l/bm6ur) | 2018 | Medical | UK | Multiple | LV | 4874 | Public | Internal holdout | Other metrics | No | 50% | 56% | 80% | 80% | 64% |
| Sun [(147)](https://paperpile.com/c/T2eW7l/2EJTn) | 2021 | Technical | Netherl&s | Short axis | LV | 325 | Public & single centre | External testing | DSC & other metrics | No | 86% | 64% | 80% | 40% | 64% |
| Tan [(148)](https://paperpile.com/c/T2eW7l/qoTj1) | 2017 | Technical | Malaysia | Short axis | LV | 1340 | Multiple public | External testing | Other metrics | No | 75% | 63% | 100% | 50% | 67% |
| Tan [(149)](https://paperpile.com/c/T2eW7l/fwx9q) | 2018 | Medical | Malaysia | Multiple | LV | 1350 | Multiple public | External testing | DSC & other metrics | No | 88% | 44% | 100% | 60% | 64% |
| Tan [(150)](https://paperpile.com/c/T2eW7l/6zN0v) | 2018 | Hybrid | Malaysia | Short axis | LV | 1346 | Multiple public | External testing | DSC & other metrics | Yes | 25% | 50% | 80% | 40% | 46% |
| Tandon [(151)](https://paperpile.com/c/T2eW7l/WxShg) | 2021 | Medical | USA | Short axis | Both ventricles | 87 | Single centre | Internal holdout | DSC & other metrics | No | 75% | 69% | 100% | 70% | 74% |
| Tao [(152)](https://paperpile.com/c/T2eW7l/OLp6M) | 2019 | Medical | Netherlands | Short axis | LV | 596 | Multicentre | External testing | DSC | No | 71% | 64% | 100% | 90% | 79% |
| Tao [(153)](https://paperpile.com/c/T2eW7l/W10HA) | 2014 | Medical | Netherlands | Short axis | LV | 50 | Single centre | Internal holdout | DSC | No | 71% | 82% | 100% | 70% | 79% |
| Tao [(154)](https://paperpile.com/c/T2eW7l/Ma1oN) | 2016 | Medical | Netherlands | NR | LA | 46 | Single centre | Internal holdout | DSC | No | 86% | 82% | 100% | 70% | 82% |
| Tarroni [(155)](https://paperpile.com/c/T2eW7l/cpqFr) | 2019 | Technical | UK | Multiple | LV | 3100 | Public | External testing | DSC | No | 75% | 75% | 100% | 70% | 77% |
| Tobon-Gomez [(156)](https://paperpile.com/c/T2eW7l/9HYMj) | 2012 | Technical | Spain | Short axis | Both ventricles | 95 | Multicentre | External testing | Other metrics | No | 63% | 69% | 100% | 70% | 72% |
| Tong [(157)](https://paperpile.com/c/T2eW7l/zf7dU) | 2019 | Technical | China | Short axis | Both ventricles | 150 | Public | Internal holdout | DSC & other metrics | No | 63% | 63% | 80% | 70% | 67% |
| Tsadok [(158)](https://paperpile.com/c/T2eW7l/wNX2R) | 2013 | Technical | Israel | Multiple | LV | 126 | Public | Internal holdout | DSC & other metrics | No | 63% | 81% | 100% | 90% | 82% |
| Tufvesson [(159)](https://paperpile.com/c/T2eW7l/iUniC) | 2016 | Hybrid | Sweden | Short axis | Myocardium | 183 | Multicentre | Internal holdout | DSC & other metrics | No | 71% | 73% | 80% | 60% | 70% |
| Ukwatta [(160)](https://paperpile.com/c/T2eW7l/f88gl) | 2014 | Technical | USA | Short axis | LV | 47 | NR | Internal holdout | DSC | No | 63% | 75% | 100% | 90% | 79% |
| Valinoti [(161)](https://paperpile.com/c/T2eW7l/EC7Uf) | 2018 | Medical | Italy | NR | LA | 26 | Single centre | Internal holdout | Other metrics | No | 71% | 73% | 80% | 70% | 73% |
| Veni [(162)](https://paperpile.com/c/T2eW7l/pbQWv) | 2013 | Technical | USA | NR | LA | 130 | NR | Internal holdout | DSC | No | 63% | 44% | 80% | 70% | 59% |
| Vesal [(163)](https://paperpile.com/c/T2eW7l/LkGoP) | 2021 | Technical | Germany | Short axis | LV | 145 | Public | Internal holdout | Other metrics | Yes | 86% | 64% | 100% | 60% | 73% |
| Vigneault [(164)](https://paperpile.com/c/T2eW7l/w7D5k) | 2018 | Technical | UK | Multiple | Four chambers | 63 | Multiple public | External testing | Other metrics | No | 38% | 50% | 100% | 70% | 59% |
| Wang [(165)](https://paperpile.com/c/T2eW7l/zfD6a) | 2020 | Medical | China | Short axis | LV | 83 | Public | Internal holdout | DSC & other metrics | Yes | 75% | 50% | 100% | 70% | 67% |
| Wang [(166)](https://paperpile.com/c/T2eW7l/7EBM6) | 2021 | Hybrid | China | Axial | Myocardium & blood pool | 20 | Public | Internal holdout | DSC | No | 63% | 56% | 100% | 40% | 59% |
| Wang [(167)](https://paperpile.com/c/T2eW7l/s5IjU) | 2021 | Medical | USA | Short axis | LV | 61 | Multiple public | External testing | DSC | No | 71% | 64% | 100% | 60% | 70% |
| Wang [(168)](https://paperpile.com/c/T2eW7l/tWoJK) | 2015 | Hybrid | China | Short axis | LV | 45 | Single centre | Internal holdout | Other metrics | No | 43% | 64% | 80% | 40% | 55% |
| Wang [(169)](https://paperpile.com/c/T2eW7l/IYkMk) | 2019 | Technical | USA | Short axis | LV & RA | 17 | Single centre | Internal holdout | DSC | No | 71% | 64% | 80% | 70% | 70% |
| Wang [(170)](https://paperpile.com/c/T2eW7l/HX5IC) | 2015 | Technical | China | Short axis | LV | 45 | Public | Internal holdout | DSC & other metrics | No | 71% | 73% | 100% | 40% | 67% |
| Wang [(171)](https://paperpile.com/c/T2eW7l/RDE8o) | 2020 | Technical | China | NR | LV | 83 | Public | Internal holdout | DSC | No | 71% | 73% | 100% | 50% | 70% |
| Wantanajittikul [(172)](https://paperpile.com/c/T2eW7l/92SLT) | 2016 | Technical | Thail& | Short axis | Myocardium | 30 | Single centre | Not reported | DSC & other metrics | No | 29% | 64% | 80% | 20% | 45% |
| Wech [(173)](https://paperpile.com/c/T2eW7l/FjAcR) | 2022 | Medical | Germany | Short axis | Both ventricles & myocardium | 567 | Multiple public | Internal holdout | DSC | Yes | 63% | 25% | 40% | 30% | 36% |
| Wei [(174)](https://paperpile.com/c/T2eW7l/hezcI) | 2013 | Technical | Singapore | Short axis | Myocardium | 20 | Single centre | Internal holdout | DSC & other metrics | No | 57% | 45% | 40% | 50% | 48% |
| Wei [(175)](https://paperpile.com/c/T2eW7l/sGF2H) | 2013 | Technical | Singapore | Multiple | LV | 21 | Single centre | External testing | DSC | No | 75% | 36% | 20% | 40% | 43% |
| Woie [(176)](https://paperpile.com/c/T2eW7l/ULoTr) | 2015 | Medical | Norway | Short axis | Scar | 41 | Single centre | Internal holdout | Other metrics | No | 75% | 19% | 20% | 30% | 33% |
| Wu [(177)](https://paperpile.com/c/T2eW7l/TsfjR) | 2021 | Technical | China | NR | LV | 195 | Multiple public | Internal holdout | Other metrics | No | 57% | 45% | 80% | 30% | 48% |
| Wu [(178)](https://paperpile.com/c/T2eW7l/rGw9t) | 2021 | Medical | China | Two chamber | Both ventricles | 55 | Public & single centre | Internal holdout | DSC | No | 75% | 31% | 20% | 80% | 51% |
| Wu [(179)](https://paperpile.com/c/T2eW7l/s9aPB) | 2020 | Technical | China | NR | LV | 45 | Public | Internal holdout | DSC & other metrics | No | 86% | 64% | 100% | 70% | 75% |
| Wu [(180)](https://paperpile.com/c/T2eW7l/B91rw) | 2021 | Medical | UK | Short axis | Myocardium | 19 | Single centre | Internal holdout | DSC & other metrics | No | 75% | 62% | 67% | 56% | 64% |
| Wu [(181)](https://paperpile.com/c/T2eW7l/V9g1J) | 2018 | Technical | USA | Coronal | NR | 19 | Single centre | Internal holdout | DSC & other metrics | No | 86% | 55% | 67% | 80% | 71% |
| Wu [(182)](https://paperpile.com/c/T2eW7l/ssvLY) | 2020 | Technical | China | Short axis | LV | 78 | Public & single centre | Internal holdout | NR | No | 86% | 58% | 50% | 78% | 69% |
| Xie [(183)](https://paperpile.com/c/T2eW7l/L7rgN) | 2020 | Technical | China | Short axis | LV | 195 | Multiple public | Internal holdout | DSC & other metrics | No | 63% | 56% | 67% | 60% | 60% |
| Xiong [(184)](https://paperpile.com/c/T2eW7l/E3FVC) | 2019 | Technical | USA | NR | LA | 60 | Single centre | Internal holdout | DSC & other metrics | No | 88% | 69% | 100% | 80% | 79% |
| Xu [(185)](https://paperpile.com/c/T2eW7l/YMSjR) | 2020 | Technical | China | Multiple | Scar | 165 | Multicentre | Internal holdout | DSC & other metrics | No | 71% | 64% | 83% | 67% | 70% |
| Xu [(186)](https://paperpile.com/c/T2eW7l/DrTNi) | 2020 | Technical | Canada | Short axis | Scar | 280 | Single centre | Internal holdout | DSC & other metrics | No | 86% | 64% | 100% | 60% | 73% |
| Xu [(187)](https://paperpile.com/c/T2eW7l/zl94c) | 2018 | Technical | China | Multiple | Scar | 165 | Single centre | Internal holdout | DSC & other metrics | No | 75% | 56% | 100% | 50% | 63% |
| Xue [(188)](https://paperpile.com/c/T2eW7l/3V040) | 2020 | Hybrid | USA | Short axis | Myocardium | 1139 | Multicentre | Internal holdout | DSC & other metrics | Yes | 88% | 44% | 100% | 80% | 69% |
| Xue [(189)](https://paperpile.com/c/T2eW7l/RrljT) | 2020 | Medical | USA | Short axis | LV | 12984 | Multicentre | Internal holdout | DSC | Yes | 88% | 69% | 100% | 60% | 74% |
| Yan [(190)](https://paperpile.com/c/T2eW7l/xYOHv) | 2019 | Technical | China | Short axis | LV | 200 | Multiple public | Internal holdout | DSC & other metrics | No | 63% | 50% | 100% | 10% | 49% |
| Yan [(191)](https://paperpile.com/c/T2eW7l/m7WJW) | 2020 | Hybrid | China | NR | LV | 150 | Multicentre | Internal holdout | DSC | No | 75% | 81% | 40% | 70% | 72% |
| Yang [(192)](https://paperpile.com/c/T2eW7l/wcy6z) | 2018 | Technical | UK | NR | Scar | 37 | Single centre | Internal holdout | DSC | No | 75% | 25% | 25% | 60% | 45% |
| Yang [(193)](https://paperpile.com/c/T2eW7l/khRIz) | 2019 | Hybrid | China | Short axis | Both ventricles & myocardium | 162 | Public & single centre | External testing | DSC & other metrics | No | 86% | 27% | 0% | 60% | 47% |
| Yang [(194)](https://paperpile.com/c/T2eW7l/S0yCd) | 2021 | Technical | China | Short axis | Both ventricles & myocardium | 150 | Public | Internal holdout | DSC & other metrics | No | 71% | 64% | 100% | 90% | 78% |
| Yang [(195)](https://paperpile.com/c/T2eW7l/WZuoU) | 2017 | Hybrid | Singapore | Short axis | LV | NR | NR | Internal holdout | DSC | No | 88% | 38% | 40% | 60% | 54% |
| Zabihollahy [(196)](https://paperpile.com/c/T2eW7l/xMHdc) | 2019 | Technical | Canada | Four chamber | Scar | 34 | Single centre | Internal holdout | DSC & other metrics | No | 88% | 69% | 100% | 70% | 77% |
| Zabihollahy [(197)](https://paperpile.com/c/T2eW7l/Y9PDA) | 2020 | Technical | Canada | Multiple | Myocardium & scar | 34 | Single centre | Internal holdout | DSC & other metrics | No | 75% | 19% | 100% | 50% | 49% |
| Zarvani [(198)](https://paperpile.com/c/T2eW7l/QIq4C) | 2021 | Technical | Iran | NR | LV | NR | Public | Internal holdout | DSC & other metrics | No | 71% | 45% | 100% | 40% | 58% |
| Zhang [(199)](https://paperpile.com/c/T2eW7l/a6irF) | 2021 | Technical | China | Short axis | Both ventricles & myocardium | 340 | Multiple public & single centre | External testing | DSC & other metrics | No | 71% | 64% | 100% | 70% | 73% |
| Zhang [(200)](https://paperpile.com/c/T2eW7l/JavOz) | 2021 | Technical | USA | Multiple | LA | 100 | Single centre | Internal holdout | DSC & other metrics | No | 88% | 69% | 100% | 70% | 76% |
| Zhang [(201)](https://paperpile.com/c/T2eW7l/us9VJ) | 2019 | Medical | China | Short axis | Scar | 299 | Single centre | Internal holdout | DSC | Yes | 88% | 54% | 75% | 70% | 69% |
| Zhao [(202)](https://paperpile.com/c/T2eW7l/tk1cX) | 2020 | Technical | China | Short axis | RA | 3 | NR | Internal holdout | DSC & other metrics | No | 88% | 50% | 60% | 80% | 67% |
| Zheng [(203)](https://paperpile.com/c/T2eW7l/7H1dS) | 2018 | Technical | France | Short axis | Both ventricles | 3980 | Multiple public | External testing | DSC & other metrics | Yes | 88% | 31% | 20% | 40% | 44% |
| Zheng [(204)](https://paperpile.com/c/T2eW7l/6TSxT) | 2014 | Medical | China | Short axis | Myocardium | 144 | NR | Internal holdout | Other metrics | No | 75% | 44% | 80% | 90% | 67% |
| Zheng [(205)](https://paperpile.com/c/T2eW7l/UvzcI) | 2015 | Medical | China | Short axis | LV | 111 | Single centre | Internal holdout | DSC | No | 75% | 44% | 60% | 60% | 56% |
| Zhu [(206)](https://paperpile.com/c/T2eW7l/9IMNw) | 2020 | Hybrid | USA | Short axis | Myocardium | 305 | Single centre | Internal holdout | DSC | No | 75% | 25% | 100% | 70% | 56% |
| Zhu [(207)](https://paperpile.com/c/T2eW7l/9HAEj) | 2013 | Technical | USA | NR | LA | 64 | Public | Internal holdout | DSC & other metrics | No | 86% | 73% | 100% | 80% | 82% |
| Zotti [(208)](https://paperpile.com/c/T2eW7l/Z8uaf) | 2019 | Technical | Canada | Short axis | Both ventricles & myocardium | 150 | Public | Internal holdout | DSC & other metrics | No | 88% | 81% | 100% | 90% | 87% |

*DSC; Dice similarity coefficient, LA; left atrium, LV; left ventricle, NR; not reported, RA; right atrium, RV; right ventricle,*

###

Compliance with the criteria of CLAIM for all included studies and the division of criteria into study description, dataset description, model description and model performance domains is shown below in Supplementary Table 2.

### **Supplementary Table 2:** Compliance with CLAIM checklist

|  | **No.** | **Criteria** | **Domain** | **Yes** | **No** |
| --- | --- | --- | --- | --- | --- |
| **Title & abstract** | | | | | |
| Title | 1 | Identification as a study of AI methodology, specifying the category of technology used (e.g., deep learning) | Study description | 90.9% | 9.1% |
| Abstract | 2 | Structured summary of study design, methods, results, and conclusions | Study description | 52.6% | 47.4% |
| **Introduction** | | | | | |
| Introduction | 3 | Scientific and clinical background, including the intended use and clinical role of the AI approach | Study description | 92.3% | 7.7% |
|  | 4 | Study objectives and hypotheses | Study description | 94.3% | 5.7% |
| **Methods** | | | | | |
| Study design | 5 | Prospective or retrospective study | Study description | 36.0% | 64.0% |
|  | 6 | Study goal, such as model creation, exploratory study, feasibility study, non-inferiority trial | Study description | 95.2% | 4.8% |
| Data sources | 7 | Data sources | Dataset description | 93.8% | 6.2% |
|  | 8 | Eligibility criteria: how, where, and when potentially eligible participants or studies were identified (e.g., symptoms, results from previous tests, inclusion in registry, patient-care setting, location, dates) | Dataset description | 74.2% | 25.8% |
|  | 9 | Data pre-processing steps | Dataset description | 93.8% | 5.7% |
|  | 10 | Selection of data subsets, if applicable | Dataset description | 92.8% | 6.7% |
|  | 11 | Definitions of data elements, with references to Common Data Elements | Dataset description | 99.5% | 0.5% |
|  | 12 | De-identification methods | Dataset description | 11.2% | 88.8% |
|  | 13 | How missing data were handled | Dataset description | 8.6% | 91.4% |
| Ground truth reference standard | 14 | Definition of ground truth reference standard, in sufficient detail to allow replication | Dataset description | 67.6% | 32.4% |
|  | 15 | Rationale for choosing the reference standard (if alternatives exist) | Dataset description | N/A | N/A |
|  | 16 | Source of ground-truth annotations; qualifications and preparation of annotators | Dataset description | 54.8% | 45.2% |
|  | 17 | Annotation tools | Dataset description | 30.6% | 69.4% |
|  | 18 | Measurement of inter- and intrarater variability; methods to mitigate variability and/or resolve discrepancies | Dataset description | 41.9% | 58.1% |
| Data Partitions | 19 | Intended sample size and how it was determined | Dataset description | 4.3% | 95.7% |
|  | 20 | How data were assigned to partitions; specify proportions | Dataset description | 89.4% | 10.6% |
|  | 21 | Level at which partitions are disjoint (e.g., image, study, patient, institution) | Dataset description | 87.0% | 13.0% |
| Model | 22 | Detailed description of model, including inputs, outputs, all intermediate layers and connections | Model description | 94.7% | 5.3% |
|  | 23 | Software libraries, frameworks, and packages | Model description | 74.2% | 25.4% |
|  | 24 | Initialization of model parameters (e.g., randomization, transfer learning) | Model description | 91.7% | 8.3% |
| Training | 25 | Details of training approach, including data augmentation, hyperparameters, number of models trained | Model description | 78.3% | 21.7% |
|  | 26 | Method of selecting the final model | Model description | 91.6% | 8.4% |
|  | 27 | Ensembling techniques, if applicable | Model description | 50.0% | 50.0% |
| Evaluation | 28 | Metrics of model performance | Model performance | 99.5% | 0.5% |
|  | 29 | Statistical measures of significance and uncertainty (e.g., confidence intervals) | Model performance | 77.5% | 22.5% |
|  | 30 | Robustness or sensitivity analysis | Model performance | 60.8% | 39.2% |
|  | 31 | Methods for explainability or interpretability (e.g., saliency maps), and how they were validated | Model performance | 64.1% | 35.9% |
|  | 32 | Validation or testing on external data | Model performance | 21.5% | 78.5% |
| **Results** | | | | | |
| Data | 33 | Flow of participants or cases, using a diagram to indicate inclusion and exclusion | Dataset description | 10.0% | 90.0% |
|  | 34 | Demographic and clinical characteristics of cases in each partition | Dataset description | 18.2% | 81.8% |
| Model performance | 35 | Performance metrics for optimal model(s) on all data partitions | Model performance | 88.9% | 11.1% |
|  | 36 | Estimates of diagnostic accuracy and their precision (such as 95% confidence intervals) | Model performance | 20.7% | 79.3% |
|  | 37 | Failure analysis of incorrectly classified cases | Model performance | 32.1% | 67.9% |
| **Discussion** | | | | | |
| Discussion | 38 | Study limitations, including potential bias, statistical uncertainty, and generalizability | Model performance | 76.1% | 23.9% |
|  | 39 | Implications for practice, including the intended use and/or clinical role | Model performance | 75.6% | 24.4% |
| **Other information** | | | | | |
| Other information | 40 | Registration number and name of registry | Study description | N/A | N/A |
|  | 41 | Where the full study protocol can be accessed | Study description | 0.0% | 100.0% |
|  | 42 | Sources of funding and other support; role of funders | Study description | 82.3% | 17.7% |

*N/A = not applicable.* Checklist adapted from Mongan et al. 2020 [(209)](https://paperpile.com/c/T2eW7l/6RTV).

Public datasets

Public datasets used in the included studies are: LV Segmentation Challenge (LVSC) in 2009 [(210)](https://paperpile.com/c/T2eW7l/rVUaE) and 2011 [(145)](https://paperpile.com/c/T2eW7l/lAFfG), LV infarct (LVIC) in 2012 [(211)](https://paperpile.com/c/T2eW7l/kWUoL), RV Segmentation Challenge (RVSC) in 2012 [(212)](https://paperpile.com/c/T2eW7l/X1jzb), Segmentation Algorithms, Theory and Applications (SATA) in 2013 [(213)](https://paperpile.com/c/T2eW7l/j6dpf), Whole-Heart and Great Vessel Segmentation from 3D Cardiovascular MRI in Congenital Heart Disease (HVSMR) in 2016 [(214)](https://paperpile.com/c/T2eW7l/NUPGh), Automated Cardiac Diagnosis Challenge (ACDC) in 2017 [(215)](https://paperpile.com/c/T2eW7l/jIfHg), Multi-Modality Whole Heart Segmentation (MM-WHS) in 2017 [(216)](https://paperpile.com/c/T2eW7l/fjdxy), Atrial Segmentation Challenge (ASC) in 2018 [(217)](https://paperpile.com/c/T2eW7l/EWRc2), Left Ventricle Full Quantification (LVQuan) in 2018 and 2019 [(218)](https://paperpile.com/c/T2eW7l/YPEGN), Multi-sequence Cardiac MR Segmentation Challenge (MS-CMRSeg) in 2019 [(219)](https://paperpile.com/c/T2eW7l/9nZc5), Evaluation of Myocardial Infarction from Delayed-Enhancement Cardiac MRI (EMIDEC) in 2020 [(220)](https://paperpile.com/c/T2eW7l/fCKtr).

### **Supplementary Table 3**: Public datasets

| **Name of dataset** | **Year** | **No. of patients** | **Cardiac chamber** | **Images** | **Main pathology** | **Source** | **Accessible** |
| --- | --- | --- | --- | --- | --- | --- | --- |
| York [(221)](https://paperpile.com/c/T2eW7l/y3WuL) | 2008 | 33 | LV | Short axis cine | Cardiomyopathy, aortic regurgitation, enlarged ventricles and ischaemia | Hospital for Sick Children, Toronto, Canada | Yes |
| LVSC [(210)](https://paperpile.com/c/T2eW7l/rVUaE) | 2009 | 45 | LV | Short axis cine | Hypertrophy, heart failure, with or without infarction | Sunnybrook Health Sciences Centre, Toronto, Canada. | Yes |
| LVSC II [(145)](https://paperpile.com/c/T2eW7l/lAFfG) | 2011 | 200 | LV | Short axis cine | Coronary artery disease and myocardial infarction | Multicenter from the DETERMINE trial (Defibrillators to Reduce Risk by Magnetic Resonance Imaging Evaluation) | No |
| LVIC [(211)](https://paperpile.com/c/T2eW7l/kWUoL) | 2012 | 30 | LV | LGE-MRI | Ischaemic cardiomyopathy | Multicentre from the Cardiac Atlas Project | Yes |
| RVSC [(212)](https://paperpile.com/c/T2eW7l/X1jzb) | 2012 | 48 | RV | Short axis cine | Myocarditis, ischaemic cardiomyopathy, suspicion of arrhythmogenic right ventricular dysplasia, dilated cardiomyopathy, hypertrophic cardiomyopathy, aortic stenosis | Rouen University Hospital, Rouen, France | Yes |
| cDEMRIS [(222)](https://paperpile.com/c/T2eW7l/Cfzt) | 2012 | 60 | LA | LGE-MRI | Atrial Fibrillation | Multicentre | Yes |
| LASC [(223)](https://paperpile.com/c/T2eW7l/5DRU) | 2013 | 30 | LA | Whole heart | Healthy | King’s College London, London, UK | No |
| SLAWT [(224)](https://paperpile.com/c/T2eW7l/ticn) | 2016 | 10 | LA | LGE-MRI | Healthy | Single centre not specified |  |
| HVSMR-I [(214)](https://paperpile.com/c/T2eW7l/NUPGh) | 2016 | 20 | Whole heart | 3D-MRI | Congenital heart defects | Boston Children’s Hospital, Boston, Massachusetts | No |
| MM-WHS [(216)](https://paperpile.com/c/T2eW7l/fjdxy) | 2017 | 60 | Whole heart | cine | Cardiac function insufficiency, cardiac edema, hypertension, sick sinus syndrome,arrhythmia, coronary atherosclerosis, aortic aneurysm, dilated cardiomyopathy aortic stenosis etc. | Single centre not specified | Yes |
| ACDC [(215)](https://paperpile.com/c/T2eW7l/jIfHg) | 2017 | 150 | LV, RV | Short axis cine | Myocardial infarction, dilated/hypertrophic cardiomyopathy,abnormal RV | University Hospital of Dijon, Dijon, France | Yes |
| LASC II [(217)](https://paperpile.com/c/T2eW7l/EWRc2) | 2018 | 154 | LA | LGE-MRI | Atrial Fibrillation | The University of Utah, Utah, USA | Yes |
| LVQuan’18 [(218)](https://paperpile.com/c/T2eW7l/YPEGN) | 2018 | 175 | LV | Short axis cine | Regional wall motion abnormalities, myocardial hypertrophy, dilated cardiomyopathy, mildly enlarged LV, atrial septal defect, LV systolic dysfunction, LAD territory ischemia, and constrictive pericarditis | London Healthcare Center and St. Joseph’s HealthCare, Ontario Canada | Yes |
| LVQuan’19 [(225)](https://paperpile.com/c/T2eW7l/pnco) | 2019 | 85 | LV | Short axis cine | Not specified. | Not specified. | Yes |
| MS-CMRS [(219)](https://paperpile.com/c/T2eW7l/9nZc5) | 2019 | 45 | LV, RV | bSSFP-MRI  LGE-MRI  T2 | Underwent cardiomyopathy | Not specified | Yes |
| OCMR [(226)](https://paperpile.com/c/T2eW7l/M9W8) | 2020 | 286 | Whole heart | cine | Non- specified | Multicentre Amazon Web Services Public Dataset | Yes |
| EMIDEC [(220)](https://paperpile.com/c/T2eW7l/fCKtr) | 2020 | 150 | LV | DE-MRI | Myocardial infarction | University Hospital of Dijon, Dijon, France | No |
| M&MS [(26)](https://paperpile.com/c/T2eW7l/6KR9T) | 2020 | 350 | Whole heart | cine | Cardiac multi-disease, different cardiomyopathies | Three countries (Spain, Germany and Canada) and different vendors (Siemens, Phillips, General Electric and Canon) | Yes |
| HVSMR-II [(227)](https://paperpile.com/c/T2eW7l/DaBZ) | 2021 | 90 | Whole heart | 3D-MRI | Congenital heart defects, involving extreme anatomical variability | Boston Children’s Hospital, Boston, Massachusetts | Yes |
| M&Ms-2 [(26)](https://paperpile.com/c/T2eW7l/6KR9T) | 2021 | 360 | Whole heart | cine | Healthy, Dilated Left Ventricle, Hypertrophic Cardiomyopathy, Congenital disease, Arrhythmogenesis, Dilated Right Ventricle, Tricuspidal Regurgitation | Multicentre, Spain | Yes |
| LAScarQS[(228)](https://paperpile.com/c/T2eW7l/AN9i) | 2022 | 194 | LA (LGE) | LGE-MRI | Atrial Fibrillation | Multicentre | Yes |

###

### ACDC; Automatic Cardiac Diagnosis Challenge, cDEMRIS; Cardiac Delayed Enhancement Segmentation Challenge, EMIDEC; automatic Evaluation of Myocardial Infarction from Delayed-Enhancement Cardiac MRI, HVSMR; Whole-Heart and Great Vessel Segmentation; LA; Left Atrium, LAScarQS, Left Atrial and Scar Quantification & Segmentation Challenge, LASC; Left Atrial Segmentation Challenge, LGE; Late Gadolinium Enhancement, LV; Left Ventricle, LVIC; Left Ventricle Infarct Challenge, LVQuan; Left Ventricle Full Quantification, LVSC; Left Ventricle Segmentation Challenge, MM-WHS; Multi-Modality Whole Heart Segmentation, M&MS; Multi-Disease, Multi-View & Multi-Center Right Ventricular Segmentation in Cardiac MRI, MS-CMRS; Multi-Sequence Cardiac MR Segmentation, RV; Right Ventricle, RVSC; Right Ventricle Segmentation Challenge, SLAWT; Segmentation of Left Atrial Wall for Thickness

###

### **Supplementary Table 4**: PRISMA checklist

| **Section and Topic** | **Item #** | **Checklist item** | **Location where item is reported** |
| --- | --- | --- | --- |
| **TITLE** | | |  |
| Title | 1 | Identify the report as a systematic review. | Page 1 |
| **ABSTRACT** | | |  |
| Abstract | 2 | See the PRISMA 2020 for Abstracts checklist. | Page 2 |
| **INTRODUCTION** | | |  |
| Rationale | 3 | Describe the rationale for the review in the context of existing knowledge. | Pages 3-4 |
| Objectives | 4 | Provide an explicit statement of the objective(s) or question(s) the review addresses. | Page 4 |
| **METHODS** | | |  |
| Eligibility criteria | 5 | Specify the inclusion and exclusion criteria for the review and how studies were grouped for the syntheses. | Page 4 |
| Information sources | 6 | Specify all databases, registers, websites, organisations, reference lists and other sources searched or consulted to identify studies. Specify the date when each source was last searched or consulted. | Page 4 |
| Search strategy | 7 | Present the full search strategies for all databases, registers and websites, including any filters and limits used. | Supplementary Materials |
| Selection process | 8 | Specify the methods used to decide whether a study met the inclusion criteria of the review, including how many reviewers screened each record and each report retrieved, whether they worked independently, and if applicable, details of automation tools used in the process. | Pages 4-5 |
| Data collection process | 9 | Specify the methods used to collect data from reports, including how many reviewers collected data from each report, whether they worked independently, any processes for obtaining or confirming data from study investigators, and if applicable, details of automation tools used in the process. | Page 5 |
| Data items | 10a | List and define all outcomes for which data were sought. Specify whether all results that were compatible with each outcome domain in each study were sought (e.g. for all measures, time points, analyses), and if not, the methods used to decide which results to collect. | Supplementary Tables 1 & 2 |
|  | 10b | List and define all other variables for which data were sought (e.g. participant and intervention characteristics, funding sources). Describe any assumptions made about any missing or unclear information. | Supplementary Tables 1 & 2 |
| Study risk of bias assessment | 11 | Specify the methods used to assess risk of bias in the included studies, including details of the tool(s) used, how many reviewers assessed each study and whether they worked independently, and if applicable, details of automation tools used in the process. | Methods |
| Effect measures | 12 | Specify for each outcome the effect measure(s) (e.g. risk ratio, mean difference) used in the synthesis or presentation of results. | Pages 5-6 |
| Synthesis methods | 13a | Describe the processes used to decide which studies were eligible for each synthesis (e.g. tabulating the study intervention characteristics and comparing against the planned groups for each synthesis (item #5)). | Page 4  Figure 1 |
|  | 13b | Describe any methods required to prepare the data for presentation or synthesis, such as handling of missing summary statistics, or data conversions. | Page 4  Figure 1 |
|  | 13c | Describe any methods used to tabulate or visually display results of individual studies and syntheses. | Figure 1  Figure 2  Supplementary Tables |
|  | 13d | Describe any methods used to synthesize results and provide a rationale for the choice(s). If meta-analysis was performed, describe the model(s), method(s) to identify the presence and extent of statistical heterogeneity, and software package(s) used. | Page 5 |
|  | 13e | Describe any methods used to explore possible causes of heterogeneity among study results (e.g. subgroup analysis, meta-regression). | N/A |
|  | 13f | Describe any sensitivity analyses conducted to assess robustness of the synthesized results. | N/A |
| Reporting bias assessment | 14 | Describe any methods used to assess risk of bias due to missing results in a synthesis (arising from reporting biases). | Methods |
| Certainty assessment | 15 | Describe any methods used to assess certainty (or confidence) in the body of evidence for an outcome. | N/A |
| **RESULTS** | | |  |
| Study selection | 16a | Describe the results of the search and selection process, from the number of records identified in the search to the number of studies included in the review, ideally using a flow diagram. | Page 5  Figure 1 |
|  | 16b | Cite studies that might appear to meet the inclusion criteria, but which were excluded, and explain why they were excluded. | No |
| Study characteristics | 17 | Cite each included study and present its characteristics. | Supplementary Table 1 |
| Risk of bias in studies | 18 | Present assessments of risk of bias for each included study. | Supplementary Table 2 |
| Results of individual studies | 19 | For all outcomes, present, for each study: (a) summary statistics for each group (where appropriate) and (b) an effect estimate and its precision (e.g. confidence/credible interval), ideally using structured tables or plots. | Figure 3  Supplementary Table 1 |
| Results of syntheses | 20a | For each synthesis, briefly summarise the characteristics and risk of bias among contributing studies. | Supplementary Table 2 |
|  | 20b | Present results of all statistical syntheses conducted. If meta-analysis was done, present for each the summary estimate and its precision (e.g. confidence/credible interval) and measures of statistical heterogeneity. If comparing groups, describe the direction of the effect. | N/A |
|  | 20c | Present results of all investigations of possible causes of heterogeneity among study results. | N/A |
|  | 20d | Present results of all sensitivity analyses conducted to assess the robustness of the synthesized results. | N/A |
| Reporting biases | 21 | Present assessments of risk of bias due to missing results (arising from reporting biases) for each synthesis assessed. | Results and Supplementary Table 2 |
| Certainty of evidence | 22 | Present assessments of certainty (or confidence) in the body of evidence for each outcome assessed. | N/A |
| **DISCUSSION** | | |  |
| Discussion | 23a | Provide a general interpretation of the results in the context of other evidence. | Pages 7-10 |
|  | 23b | Discuss any limitations of the evidence included in the review. | Pages 7-10 |
|  | 23c | Discuss any limitations of the review processes used. | Page 9 |
|  | 23d | Discuss implications of the results for practice, policy, and future research. | Page 9 |
| **OTHER INFORMATION** | | |  |
| Registration and protocol | 24a | Provide registration information for the review, including register name and registration number, or state that the review was not registered. | Page 5 |
|  | 24b | Indicate where the review protocol can be accessed, or state that a protocol was not prepared. | Page 4 |
|  | 24c | Describe and explain any amendments to information provided at registration or in the protocol. | N/A |
| Support | 25 | Describe sources of financial or non-financial support for the review, and the role of the funders or sponsors in the review. | Page 1 |
| Competing interests | 26 | Declare any competing interests of review authors. | Page 1 |
| Availability of data, code and other materials | 27 | Report which of the following are publicly available and where they can be found: template data collection forms; data extracted from included studies; data used for all analyses; analytic code; any other materials used in the review. | N/A |

# References

1. [Abdelaziz D, Saïd M, Amine CM. Automatic Segmentation of the Right Ventricle by Active Shape Model and a Distance Transform. *Journal of Medical Imaging and Health Informatics* (2015) 5:27–35. doi:](http://paperpile.com/b/T2eW7l/m9koC) [10.1166/jmihi.2015.1353](http://dx.doi.org/10.1166/jmihi.2015.1353)

2. [Abdelrauof D, Essam M, Elattar M. Light-Weight Localization and Scale-Independent Multi-gate UNET Segmentation of Left and Right Ventricles in MRI Images. *Cardiovasc Eng Technol* (2021) doi:](http://paperpile.com/b/T2eW7l/qPLOM) [10.1007/s13239-021-00591-2](http://dx.doi.org/10.1007/s13239-021-00591-2)

3. [Abdeltawab H, Khalifa F, Taher F, Alghamdi NS, Ghazal M, Beache G, Mohamed T, Keynton R, El-Baz A. A deep learning-based approach for automatic segmentation and quantification of the left ventricle from cardiac cine MR images. *Computerized Medical Imaging and Graphics* (2020) 81:101717. doi:](http://paperpile.com/b/T2eW7l/EAj2v) [10.1016/j.compmedimag.2020.101717](http://dx.doi.org/10.1016/j.compmedimag.2020.101717)

4. [Afshin M, Ben Ayed I, Punithakumar K, Law M, Islam A, Goela A, Peters T, Li S. Regional Assessment of Cardiac Left Ventricular Myocardial Function via MRI Statistical Features. *IEEE Transactions on Medical Imaging* (2014) 33:481–494. doi:](http://paperpile.com/b/T2eW7l/4wm9i) [10.1109/tmi.2013.2287793](http://dx.doi.org/10.1109/tmi.2013.2287793)

5. [Alandejani F, Alabed S, Garg P, Goh ZM, Karunasaagarar K, Sharkey M, Salehi M, Aldabbagh Z, Dwivedi K, Mamalakis M, et al. Training and clinical testing of artificial intelligence derived right atrial cardiovascular magnetic resonance measurements. *Journal of Cardiovascular Magnetic Resonance* (2022) 24: doi:](http://paperpile.com/b/T2eW7l/eINfB) [10.1186/s12968-022-00855-3](http://dx.doi.org/10.1186/s12968-022-00855-3)

6. [Alba X, Pereanez M, Hoogendoorn C, Swift AJ, Wild JM, Frangi AF, Lekadir K. An Algorithm for the Segmentation of Highly Abnormal Hearts Using a Generic Statistical Shape Model. *IEEE Transactions on Medical Imaging* (2016) 35:845–859. doi:](http://paperpile.com/b/T2eW7l/owg5Z) [10.1109/tmi.2015.2497906](http://dx.doi.org/10.1109/tmi.2015.2497906)

7. [Albà X, Lekadir K, Pereañez M, Medrano-Gracia P, Young AA, Frangi AF. Automatic initialization and quality control of large-scale cardiac MRI segmentations. *Medical Image Analysis* (2018) 43:129–141. doi:](http://paperpile.com/b/T2eW7l/L0ad2) [10.1016/j.media.2017.10.001](http://dx.doi.org/10.1016/j.media.2017.10.001)

8. [Ammar A, Bouattane O, Youssfi M. Automatic cardiac cine MRI segmentation and heart disease classification. *Comput Med Imaging Graph* (2021) 88:101864.](http://paperpile.com/b/T2eW7l/otstX)

9. [Ammar M, Mahmoudi S, Chikh MA, Abbou A. Endocardial border detection in cardiac magnetic resonance images using level set method. *J Digit Imaging* (2012) 25:294–306.](http://paperpile.com/b/T2eW7l/EV2uj)

10. [Ankenbrand MJ, Lohr D, Schlötelburg W, Reiter T, Wech T, Schreiber LM. Deep learning‐based cardiac cine segmentation: Transfer learning application to 7T ultrahigh‐field MRI. *Magnetic Resonance in Medicine* (2021) 86:2179–2191. doi:](http://paperpile.com/b/T2eW7l/JHS9D) [10.1002/mrm.28822](http://dx.doi.org/10.1002/mrm.28822)

11. [Atehortúa A, Zuluaga MA, García JD, Romero E. Automatic segmentation of right ventricle in cardiac cine MR images using a saliency analysis. *Medical Physics* (2016) 43:6270–6281. doi:](http://paperpile.com/b/T2eW7l/iVFEC) [10.1118/1.4966133](http://dx.doi.org/10.1118/1.4966133)

12. [Attar R, Pereañez M, Gooya A, Albà X, Zhang L, de Vila MH, Lee AM, Aung N, Lukaschuk E, Sanghvi MM, et al. Quantitative CMR population imaging on 20,000 subjects of the UK Biobank imaging study: LV/RV quantification pipeline and its evaluation. *Med Image Anal* (2019) 56:26–42.](http://paperpile.com/b/T2eW7l/6dyDM)

13. [Augusto JB, Davies RH, Bhuva AN, Knott KD, Seraphim A, Alfarih M, Lau C, Hughes RK, Lopes LR, Shiwani H, et al. Diagnosis and risk stratification in hypertrophic cardiomyopathy using machine learning wall thickness measurement: a comparison with human test-retest performance. *The Lancet Digital Health* (2021) 3:e20–e28.](http://paperpile.com/b/T2eW7l/iHjgh)

14. [Avendi MR, Kheradvar A, Jafarkhani H. A combined deep-learning and deformable-model approach to fully automatic segmentation of the left ventricle in cardiac MRI. *Med Image Anal* (2016) 30:108–119.](http://paperpile.com/b/T2eW7l/3oHVF)

15. [Avendi MR, Kheradvar A, Jafarkhani H. Automatic segmentation of the right ventricle from cardiac MRI using a learning-based approach. *Magn Reson Med* (2017) 78:2439–2448.](http://paperpile.com/b/T2eW7l/lanz9)

16. [Bai W, Sinclair M, Tarroni G, Oktay O, Rajchl M, Vaillant G, Lee AM, Aung N, Lukaschuk E, Sanghvi MM, et al. Automated cardiovascular magnetic resonance image analysis with fully convolutional networks. *J Cardiovasc Magn Reson* (2018) 20:65.](http://paperpile.com/b/T2eW7l/OKlUp)

17. [Barba-J L, Escalante-Ramírez B, Venegas EV, Cosío FA. A 3D Hermite-based multiscale local active contour method with elliptical shape constraints for segmentation of cardiac MR and CT volumes. *Medical & Biological Engineering & Computing* (2018) 56:833–851. doi:](http://paperpile.com/b/T2eW7l/ZZvRU) [10.1007/s11517-017-1732-9](http://dx.doi.org/10.1007/s11517-017-1732-9)

18. [Bartoli A, Fournel J, Bentatou Z, Habib G, Lalande A, Bernard M, Boussel L, Pontana F, Dacher J-N, Ghattas B, et al. Deep Learning-based Automated Segmentation of Left Ventricular Trabeculations and Myocardium on Cardiac MR Images: A Feasibility Study. *Radiol Artif Intell* (2021) 3:e200021.](http://paperpile.com/b/T2eW7l/yAJi3)

19. [Beache GM, Khalifa F, El-Baz A, Gimel’farb G. Fully automated framework for the analysis of myocardial first-pass perfusion MR images. *Med Phys* (2014) 41:102305.](http://paperpile.com/b/T2eW7l/vc9Hm)

20. [Bhatt N, Ramanan V, Gunraj H, Guo F, Biswas L, Qi X, Roifman I, Wright GA, Ghugre NR. Technical Note: Fully automatic segmental relaxometry (FASTR) for cardiac magnetic resonance T1 mapping. *Med Phys* (2021) 48:1815–1822.](http://paperpile.com/b/T2eW7l/XlCtH)

21. [Bhuva A, Bai W, Lau C, Davies R, Ye Y, Bulluck H, McAlindon E, Culotta V, Swoboda P, Captur G, et al. A Multicenter, Scan-Rescan, Human and Machine Learning CMR Study to Test Generalizability and Precision in Imaging Biomarker Analysis. *Circ Cardiovasc Imaging* (2019) 12:e009214.](http://paperpile.com/b/T2eW7l/axJMK)

22. [Borra D, Andalò A, Paci M, Fabbri C, Corsi C. A fully automated left atrium segmentation approach from late gadolinium enhanced magnetic resonance imaging based on a convolutional neural network. *Quant Imaging Med Surg* (2020) 10:1894–1907.](http://paperpile.com/b/T2eW7l/zJQYB)

23. [Brahim K, Qayyum A, Lalande A, Boucher A, Sakly A, Meriaudeau F. A 3D Network Based Shape Prior for Automatic Myocardial Disease Segmentation in Delayed-Enhancement MRI. *IRBM* (2021) 42:424–434.](http://paperpile.com/b/T2eW7l/1jZpl)

24. [Brodoefel H, Tsiflikas I, Kramer U, Lang N, Reimann A, Burgstahler C, Claussen CD, Heuschmid M. Accuracy of automated attenuation-based 3-dimensional segmentation: in the analysis of left ventricular function compared with magnetic resonance imaging. *Tex Heart Inst J* (2012) 39:36–43.](http://paperpile.com/b/T2eW7l/9KfTq)

25. [Budai A, Suhai FI, Csorba K, Toth A, Szabo L, Vago H, Merkely B. Fully automatic segmentation of right and left ventricle on short-axis cardiac MRI images. *Comput Med Imaging Graph* (2020) 85:101786.](http://paperpile.com/b/T2eW7l/7Vsao)

26. [Campello VM, Gkontra P, Izquierdo C, Martin-Isla C, Sojoudi A, Full PM, Maier-Hein K, Zhang Y, He Z, Ma J, et al. Multi-Centre, Multi-Vendor and Multi-Disease Cardiac Segmentation: The M&Ms Challenge. *IEEE Trans Med Imaging* (2021) 40:3543–3554.](http://paperpile.com/b/T2eW7l/6KR9T)

27. [Carbajal-Degante E, Avendaño S, Ledesma L, Olveres J, Vallejo E, Escalante-Ramirez B. A multiphase texture-based model of active contours assisted by a convolutional neural network for automatic CT and MRI heart ventricle segmentation. *Comput Methods Programs Biomed* (2021) 211:106373.](http://paperpile.com/b/T2eW7l/vOKUi)

28. [Cardenas R, Curiale AH, Mato G. Left ventricle segmentation using a Bayesian approach with distance dependent shape priors. *Biomed Phys Eng Express* (2020) 6:045013.](http://paperpile.com/b/T2eW7l/Z2t0w)

29. [Carminati MC, Chiara Carminati M, Boniotti C, Fusini L, Andreini D, Pontone G, Pepi M, Caiani EG. Comparison of Image Processing Techniques for Nonviable Tissue Quantification in Late Gadolinium Enhancement Cardiac Magnetic Resonance Images. *Journal of Thoracic Imaging* (2016) 31:168–176. doi:](http://paperpile.com/b/T2eW7l/AYQxe) [10.1097/rti.0000000000000206](http://dx.doi.org/10.1097/rti.0000000000000206)

30. [Chang Y, Jung C. Automatic cardiac MRI segmentation and permutation-invariant pathology classification using deep neural networks and point clouds. *Neurocomputing* (2020) 418:270–279.](http://paperpile.com/b/T2eW7l/j7xCb)

31. [Chen V, Barker AJ, Golan R, Scott MB, Huh H, Wei Q, Sojoudi A, Markl M. Effect of age and sex on fully automated deep learning assessment of left ventricular function, volumes, and contours in cardiac magnetic resonance imaging. *Int J Cardiovasc Imaging* (2021) 37:3539–3547.](http://paperpile.com/b/T2eW7l/LnM7y)

32. [Chen J, Zhang H, Zhang W, Du X, Zhang Y, Li S. Correlated Regression Feature Learning for Automated Right Ventricle Segmentation. *IEEE Journal of Translational Engineering in Health and Medicine* (2018) 6:1–10. doi:](http://paperpile.com/b/T2eW7l/TNoPT) [10.1109/jtehm.2018.2804947](http://dx.doi.org/10.1109/jtehm.2018.2804947)

33. [Chenoune Y, Pellot-Barakat C, Constantinides C, El Berbari R, Lefort M, Roullot E, Mousseaux E, Frouin F. Methodology for jointly assessing myocardial infarct extent and regional contraction in 3-D CMRI. *IEEE Trans Biomed Eng* (2012) 59:2650–2659.](http://paperpile.com/b/T2eW7l/atS9s)

34. [Cui H, Yuwen C, Jiang L, Xia Y, Zhang Y. Multiscale attention guided U-Net architecture for cardiac segmentation in short-axis MRI images. *Comput Methods Programs Biomed* (2021) 206:106142.](http://paperpile.com/b/T2eW7l/cttKC)

35. [Curiale AH, Colavecchia FD, Mato G. Automatic quantification of the LV function and mass: A deep learning approach for cardiovascular MRI. *Comput Methods Programs Biomed* (2019) 169:37–50.](http://paperpile.com/b/T2eW7l/igrt5)

36. [Davies RH, Augusto JB, Bhuva A, Xue H, Treibel TA, Ye Y, Hughes RK, Bai W, Lau C, Shiwani H, et al. Precision measurement of cardiac structure and function in cardiovascular magnetic resonance using machine learning. *J Cardiovasc Magn Reson* (2022) 24:16.](http://paperpile.com/b/T2eW7l/o5fkD)

37. [Daviller C, Grenier T, Ratiney H, Sdika M, Croisille P, Viallon M. Automatic myocardial ischemic lesion detection on magnetic resonance perfusion weighted imaging prior perfusion quantification: A pre-modeling strategy. *Computers in Biology and Medicine* (2019) 110:108–119. doi:](http://paperpile.com/b/T2eW7l/iOvJT) [10.1016/j.compbiomed.2019.05.001](http://dx.doi.org/10.1016/j.compbiomed.2019.05.001)

38. [Dharanibai G, Raina JP. Automatic segmentation of left ventricle endocardium from cardiac MR images using active contours driven by local and global intensity fitting energy. *International Journal of Medical Engineering and Informatics* (2014) 6:115. doi:](http://paperpile.com/b/T2eW7l/jZAO7) [10.1504/ijmei.2014.060247](http://dx.doi.org/10.1504/ijmei.2014.060247)

39. [Diller G-P, Vahle J, Radke R, Vidal MLB, Fischer AJ, Bauer UMM, Sarikouch S, Berger F, Beerbaum P, Baumgartner H, et al. Utility of deep learning networks for the generation of artificial cardiac magnetic resonance images in congenital heart disease. *BMC Medical Imaging* (2020) 20: doi:](http://paperpile.com/b/T2eW7l/C1xu2) [10.1186/s12880-020-00511-1](http://dx.doi.org/10.1186/s12880-020-00511-1)

40. [Do HP, Guo Y, Yoon AJ, Nayak KS. Accuracy, uncertainty, and adaptability of automatic myocardial ASL segmentation using deep CNN. *Magn Reson Med* (2020) 83:1863–1874.](http://paperpile.com/b/T2eW7l/Uf8hb)

41. [Dreijer JF, Herbst BM, du Preez JA. Left ventricular segmentation from MRI datasets with edge modelling conditional random fields. *BMC Med Imaging* (2013) 13:24.](http://paperpile.com/b/T2eW7l/hxJIt)

42. [Du X, Song Y, Liu Y, Zhang Y, Liu H, Chen B, Li S. An integrated deep learning framework for joint segmentation of blood pool and myocardium. *Med Image Anal* (2020) 62:101685.](http://paperpile.com/b/T2eW7l/N1NXv)

43. [Du X, Xu X, Liu H, Li S. TSU-net: Two-stage multi-scale cascade and multi-field fusion U-net for right ventricular segmentation. *Comput Med Imaging Graph* (2021) 93:101971.](http://paperpile.com/b/T2eW7l/e9i8c)

44. [Du X, Tang R, Yin S, Zhang Y, Li S. Direct Segmentation-Based Full Quantification for Left Ventricle via Deep Multi-Task Regression Learning Network. *IEEE Journal of Biomedical and Health Informatics* (2019) 23:942–948. doi:](http://paperpile.com/b/T2eW7l/Y5e9Y) [10.1109/jbhi.2018.2879188](http://dx.doi.org/10.1109/jbhi.2018.2879188)

45. [Du X, Yin S, Tang R, Liu Y, Song Y, Zhang Y, Liu H, Li S. Segmentation and visualization of left atrium through a unified deep learning framework. *International Journal of Computer Assisted Radiology and Surgery* (2020) 15:589–600. doi:](http://paperpile.com/b/T2eW7l/sLMS6) [10.1007/s11548-020-02128-9](http://dx.doi.org/10.1007/s11548-020-02128-9)

46. [El-Rewaidy H, Fahmy AS, Khalifa AM, Ibrahim E-SH. Multiple two-dimensional active shape model framework for right ventricular segmentation. *Magn Reson Imaging* (2022) 85:177–185.](http://paperpile.com/b/T2eW7l/zrCgE)

47. [Eslami A, Karamalis A, Katouzian A, Navab N. Segmentation by retrieval with guided random walks: Application to left ventricle segmentation in MRI. *Medical Image Analysis* (2013) 17:236–253. doi:](http://paperpile.com/b/T2eW7l/Vvbxb) [10.1016/j.media.2012.10.005](http://dx.doi.org/10.1016/j.media.2012.10.005)

48. [Fadil H, Totman JJ, Hausenloy DJ, Ho H-H, Joseph P, Low AF-H, Mark Richards A, Chan MY, Marchesseau S. A deep learning pipeline for automatic analysis of multi-scan cardiovascular magnetic resonance. *Journal of Cardiovascular Magnetic Resonance* (2021) 23: doi:](http://paperpile.com/b/T2eW7l/oMDAU) [10.1186/s12968-020-00695-z](http://dx.doi.org/10.1186/s12968-020-00695-z)

49. [Fahmy AS, El-Rewaidy H, Nezafat M, Nakamori S, Nezafat R. Automated analysis of cardiovascular magnetic resonance myocardial native T1 mapping images using fully convolutional neural networks. *J Cardiovasc Magn Reson* (2019) 21:7.](http://paperpile.com/b/T2eW7l/lZTJ3)

50. [Fahmy AS, Rowin EJ, Chan RH, Manning WJ, Maron MS, Nezafat R. Improved Quantification of Myocardium Scar in Late Gadolinium Enhancement Images: Deep Learning Based Image Fusion Approach. *J Magn Reson Imaging* (2021) 54:303–312.](http://paperpile.com/b/T2eW7l/bNklm)

51. [Fahmy AS, Neisius U, Chan RH, Rowin EJ, Manning WJ, Maron MS, Nezafat R. Three-dimensional Deep Convolutional Neural Networks for Automated Myocardial Scar Quantification in Hypertrophic Cardiomyopathy: A Multicenter Multivendor Study. *Radiology* (2020) 294:52–60.](http://paperpile.com/b/T2eW7l/IVrsN)

52. [Farrag NA, Lochbihler A, White JA, Ukwatta E. Evaluation of fully automated myocardial segmentation techniques in native and contrast‐enhanced T1‐mapping cardiovascular magnetic resonance images using fully convolutional neural networks. *Medical Physics* (2021) 48:215–226. doi:](http://paperpile.com/b/T2eW7l/3AN4H) [10.1002/mp.14574](http://dx.doi.org/10.1002/mp.14574)

53. [Yang F, Yang X, Teo SK, Lee G, Zhong L, Tan RS, Su Y. Multi-dimensional proprio-proximus machine learning for assessment of myocardial infarction. *Comput Med Imaging Graph* (2018) 70:63–72.](http://paperpile.com/b/T2eW7l/4uNRv)

54. [Feng C, Zhang S, Zhao D, Li C. Simultaneous extraction of endocardial and epicardial contours of the left ventricle by distance regularized level sets. *Med Phys* (2016) 43:2741–2755.](http://paperpile.com/b/T2eW7l/Beai5)

55. [Ferreira PF, Martin RR, Scott AD, Khalique Z, Yang G, Nielles‐Vallespin S, Pennell DJ, Firmin DN. Automating in vivo cardiac diffusion tensor postprocessing with deep learning–based segmentation. *Magnetic Resonance in Medicine* (2020) 84:2801–2814. doi:](http://paperpile.com/b/T2eW7l/pd1LO) [10.1002/mrm.28294](http://dx.doi.org/10.1002/mrm.28294)

56. [Freling HG, van Wijk K, Jaspers K, Pieper PG, Vermeulen KM, van Swieten JM, Willems TP. Impact of right ventricular endocardial trabeculae on volumes and function assessed by CMR in patients with tetralogy of Fallot. *Int J Cardiovasc Imaging* (2013) 29:625–631.](http://paperpile.com/b/T2eW7l/SJkaA)

57. [Gao H, Kadir K, Payne AR, Soraghan J, Berry C. Highly automatic quantification of myocardial oedema in patients with acute myocardial infarction using bright blood T2-weighted CMR. *J Cardiovasc Magn Reson* (2013) 15:28.](http://paperpile.com/b/T2eW7l/1ZHEQ)

58. [Ghadimi S, Auger DA, Feng X, Sun C, Meyer CH, Bilchick KC, Cao JJ, Scott AD, Oshinski JN, Ennis DB, et al. Fully-automated global and segmental strain analysis of DENSE cardiovascular magnetic resonance using deep learning for segmentation and phase unwrapping. *Journal of Cardiovascular Magnetic Resonance* (2021) 23: doi:](http://paperpile.com/b/T2eW7l/PPc84) [10.1186/s12968-021-00712-9](http://dx.doi.org/10.1186/s12968-021-00712-9)

59. [Giannakidis A, Nyktari E, Keegan J, Pierce I, Horduna IS, Haldar S, Pennell DJ, Mohiaddin R, Wong T, Firmin DN. Rapid automatic segmentation of abnormal tissue in late gadolinium enhancement cardiovascular magnetic resonance images for improved management of long-standing persistent atrial fibrillation. *BioMedical Engineering OnLine* (2015) 14: doi:](http://paperpile.com/b/T2eW7l/PNYMS) [10.1186/s12938-015-0083-8](http://dx.doi.org/10.1186/s12938-015-0083-8)

60. [Gonzales RA, Seemann F, Lamy J, Arvidsson PM, Heiberg E, Murray V, Peters DC. Automated left atrial time-resolved segmentation in MRI long-axis cine images using active contours. *BMC Med Imaging* (2021) 21:101.](http://paperpile.com/b/T2eW7l/oi1ev)

61. [Goyal N, Mor-Avi V, Volpato V, Narang A, Wang S, Salerno M, Lang RM, Patel AR. Machine learning based quantification of ejection and filling parameters by fully automated dynamic measurement of left ventricular volumes from cardiac magnetic resonance images. *Magn Reson Imaging* (2020) 67:28–32.](http://paperpile.com/b/T2eW7l/zvYON)

62. [Goyal A. Image-based clustering and connected component labeling for rapid automated left and right ventricular endocardial volume extraction and segmentation in full cardiac cycle multi-frame MRI images of cardiac patients. *Medical & Biological Engineering & Computing* (2019) 57:1213–1228. doi:](http://paperpile.com/b/T2eW7l/a3bDT) [10.1007/s11517-019-01952-9](http://dx.doi.org/10.1007/s11517-019-01952-9)

63. [Guo F, Ng M, Goubran M, Petersen SE, Piechnik SK, Neubauer S, Wright G. Improving cardiac MRI convolutional neural network segmentation on small training datasets and dataset shift: A continuous kernel cut approach. *Med Image Anal* (2020) 61:101636.](http://paperpile.com/b/T2eW7l/9z5pN)

64. [Gupta V, Bustamante M, Fredriksson A, Carlhäll C-J, Ebbers T. Improving left ventricular segmentation in four-dimensional flow MRI using intramodality image registration for cardiac blood flow analysis. *Magnetic Resonance in Medicine* (2018) 79:554–560. doi:](http://paperpile.com/b/T2eW7l/PnAZ3) [10.1002/mrm.26674](http://dx.doi.org/10.1002/mrm.26674)

65. [Hajiaghayi M, Groves EM, Jafarkhani H, Kheradvar A. A 3-D Active Contour Method for Automated Segmentation of the Left Ventricle From Magnetic Resonance Images. *IEEE Trans Biomed Eng* (2017) 64:134–144.](http://paperpile.com/b/T2eW7l/KAXmC)

66. [Hann E, Popescu IA, Zhang Q, Gonzales RA, Barutçu A, Neubauer S, Ferreira VM, Piechnik SK. Deep neural network ensemble for on-the-fly quality control-driven segmentation of cardiac MRI T1 mapping. *Med Image Anal* (2021) 71:102029.](http://paperpile.com/b/T2eW7l/Vl6du)

67. [Hautvast GLTF, Hautvast GLT, Salton CJ, Chuang ML, Breeuwer M, O’Donnell CJ, Manning WJ. Accurate computer-aided quantification of left ventricular parameters: Experience in 1555 cardiac magnetic resonance studies from the Framingham Heart Study. *Magnetic Resonance in Medicine* (2012) 67:1478–1486. doi:](http://paperpile.com/b/T2eW7l/6PAWG) [10.1002/mrm.23127](http://dx.doi.org/10.1002/mrm.23127)

68. [He Y, Qin W, Wu Y, Zhang M, Yang Y, Liu X, Zheng H, Liang D, Hu Z. Automatic left ventricle segmentation from cardiac magnetic resonance images using a capsule network. *J Xray Sci Technol* (2020) 28:541–553.](http://paperpile.com/b/T2eW7l/9cJ1R)

69. [Heidenreich JF, Gassenmaier T, Ankenbrand MJ, Bley TA, Wech T. Self-configuring nnU-net pipeline enables fully automatic infarct segmentation in late enhancement MRI after myocardial infarction. *Eur J Radiol* (2021) 141:109817.](http://paperpile.com/b/T2eW7l/6RvOW)

70. [Ho N, Kim Y-C. Evaluation of transfer learning in deep convolutional neural network models for cardiac short axis slice classification. *Sci Rep* (2021) 11:1839.](http://paperpile.com/b/T2eW7l/HM0JA)

71. [Hu H, Pan N, Wang J, Yin T, Ye R. Automatic segmentation of left ventricle from cardiac MRI via deep learning and region constrained dynamic programming. *Neurocomputing* (2019) 347:139–148.](http://paperpile.com/b/T2eW7l/3FvUJ)

72. [Hu H, Gao Z, Liu L, Liu H, Gao J, Xu S, Li W, Huang L. Automatic segmentation of the left ventricle in cardiac MRI using local binary fitting model and dynamic programming techniques. *PLoS One* (2014) 9:e114760.](http://paperpile.com/b/T2eW7l/Cdb8G)

73. [Hu H, Liu H, Gao Z, Huang L. Hybrid segmentation of left ventricle in cardiac MRI using gaussian-mixture model and region restricted dynamic programming. *Magnetic Resonance Imaging* (2013) 31:575–584. doi:](http://paperpile.com/b/T2eW7l/e7OYd) [10.1016/j.mri.2012.10.004](http://dx.doi.org/10.1016/j.mri.2012.10.004)

74. [Huang H-H, Huang C-Y, Chen C-N, Wang Y-W, Huang T-Y. Automatic regional analysis of myocardial native T1 values: left ventricle segmentation and AHA parcellations. *Int J Cardiovasc Imaging* (2018) 34:131–140.](http://paperpile.com/b/T2eW7l/O6cH7)

75. [Isensee F, Jaeger PF, Full PM, Wolf I, Engelhardt S, Maier-Hein KH. Automatic Cardiac Disease Assessment on cine-MRI via Time-Series Segmentation and Domain Specific Features. *Statistical Atlases and Computational Models of the Heart. ACDC and MMWHS Challenges*. Springer International Publishing (2018). p. 120–129](http://paperpile.com/b/T2eW7l/KYCwI)

76. [Kadir K, Gao H, Payne A, Soraghan J, Berry C. LV wall segmentation using the variational level set method (LSM) with additional shape constraint for oedema quantification. *Phys Med Biol* (2012) 57:6007–6023.](http://paperpile.com/b/T2eW7l/2Xoxu)

77. [Karr J, Cohen M, McQuiston SA, Poorsala T, Malozzi C. Validation of a deep-learning semantic segmentation approach to fully automate MRI-based left-ventricular deformation analysis in cardiotoxicity. *The British Journal of Radiology* (2021) 94:20201101. doi:](http://paperpile.com/b/T2eW7l/o2GOK) [10.1259/bjr.20201101](http://dx.doi.org/10.1259/bjr.20201101)

78. [Kar BJ, Cohen MV, McQuiston SP, Malozzi CM. A deep-learning semantic segmentation approach to fully automated MRI-based left-ventricular deformation analysis in cardiotoxicity. *Magnetic Resonance Imaging* (2021) 78:127–139. doi:](http://paperpile.com/b/T2eW7l/QEuBE) [10.1016/j.mri.2021.01.005](http://dx.doi.org/10.1016/j.mri.2021.01.005)

79. [Kar J, Cohen MV, McQuiston SA, Malozzi CM. Comprehensive enhanced methodology of an MRI-based automated left-ventricular chamber quantification algorithm and validation in chemotherapy-related cardiotoxicity. *Journal of Medical Imaging* (2020) 7: doi:](http://paperpile.com/b/T2eW7l/SGAr9) [10.1117/1.jmi.7.6.064002](http://dx.doi.org/10.1117/1.jmi.7.6.064002)

80. [Khalifa F, Beache GM, Gimel’farb G, Giridharan GA, El-Baz A. Accurate automatic analysis of cardiac cine images. *IEEE Trans Biomed Eng* (2012) 59:445–455.](http://paperpile.com/b/T2eW7l/rLvWj)

81. [Khamechian M-B, Saadatmand-Tarzjan M. FoCA: A new framework of coupled geometric active contours for segmentation of 3D cardiac magnetic resonance images. *Magnetic Resonance Imaging* (2018) 51:51–60. doi:](http://paperpile.com/b/T2eW7l/cROpB) [10.1016/j.mri.2018.04.011](http://dx.doi.org/10.1016/j.mri.2018.04.011)

82. [Khened M, Kollerathu VA, Krishnamurthi G. Fully convolutional multi-scale residual DenseNets for cardiac segmentation and automated cardiac diagnosis using ensemble of classifiers. *Medical Image Analysis* (2019) 51:21–45. doi:](http://paperpile.com/b/T2eW7l/hgFAH) [10.1016/j.media.2018.10.004](http://dx.doi.org/10.1016/j.media.2018.10.004)

83. [Kim Y-C, Kim KR, Choe YH. Automatic myocardial segmentation in dynamic contrast enhanced perfusion MRI using Monte Carlo dropout in an encoder-decoder convolutional neural network. *Computer Methods and Programs in Biomedicine* (2020) 185:105150. doi:](http://paperpile.com/b/T2eW7l/F5FMS) [10.1016/j.cmpb.2019.105150](http://dx.doi.org/10.1016/j.cmpb.2019.105150)

84. [Kim Y-C, Kim KR, Choi K, Kim M, Chung Y, Choe YH. EVCMR: A tool for the quantitative evaluation and visualization of cardiac MRI data. *Computers in Biology and Medicine* (2019) 111:103334. doi:](http://paperpile.com/b/T2eW7l/SIuC4) [10.1016/j.compbiomed.2019.103334](http://dx.doi.org/10.1016/j.compbiomed.2019.103334)

85. [Koehler S, Hussain T, Blair Z, Huffaker T, Ritzmann F, Tandon A, Pickardt T, Sarikouch S, Latus H, Greil G, et al. Unsupervised Domain Adaptation From Axial to Short-Axis Multi-Slice Cardiac MR Images by Incorporating Pretrained Task Networks. *IEEE Transactions on Medical Imaging* (2021) 40:2939–2953. doi:](http://paperpile.com/b/T2eW7l/M8iMi) [10.1109/tmi.2021.3052972](http://dx.doi.org/10.1109/tmi.2021.3052972)

86. [Ramana, Kumar. Left Ventricle Of Cardiovascular Image Segmentation Using T-Segnet Hybrid And Extended Buffalo Optimization. *Eur J Mol Clin Med*](http://paperpile.com/b/T2eW7l/Rukm6) <https://ejmcm.com/article_4561.html>

87. [Kurzendorfer T, Forman C, Schmidt M, Tillmanns C, Maier A, Brost A. Fully automatic segmentation of left ventricular anatomy in 3-D LGE-MRI. *Comput Med Imaging Graph* (2017) 59:13–27.](http://paperpile.com/b/T2eW7l/lGryz)

88. [Lebenberg J, Buvat I, Lalande A, Clarysse P, Casta C, Cochet A, Constantinides C, Cousty J, de Cesare A, Jehan-Besson S, et al. Nonsupervised Ranking of Different Segmentation Approaches: Application to the Estimation of the Left Ventricular Ejection Fraction From Cardiac Cine MRI Sequences. *IEEE Transactions on Medical Imaging* (2012) 31:1651–1660. doi:](http://paperpile.com/b/T2eW7l/pKNRH) [10.1109/tmi.2012.2201737](http://dx.doi.org/10.1109/tmi.2012.2201737)

89. [Li J, Yu ZL, Gu Z, Liu H, Li Y. Dilated-Inception Net: Multi-Scale Feature Aggregation for Cardiac Right Ventricle Segmentation. *IEEE Transactions on Biomedical Engineering* (2019) 66:3499–3508. doi:](http://paperpile.com/b/T2eW7l/Z64yY) [10.1109/tbme.2019.2906667](http://dx.doi.org/10.1109/tbme.2019.2906667)

90. [Li L, Wu F, Yang G, Xu L, Wong T, Mohiaddin R, Firmin D, Keegan J, Zhuang X. Atrial scar quantification via multi-scale CNN in the graph-cuts framework. *Medical Image Analysis* (2020) 60:101595. doi:](http://paperpile.com/b/T2eW7l/sxaaf) [10.1016/j.media.2019.101595](http://dx.doi.org/10.1016/j.media.2019.101595)

91. [Liao F, Chen X, Hu X, Song S. Estimation of the Volume of the Left Ventricle From MRI Images Using Deep Neural Networks. *IEEE Trans Cybern* (2019) 49:495–504.](http://paperpile.com/b/T2eW7l/esyVm)

92. [Lin A, Wu J, Yang X. A data augmentation approach to train fully convolutional networks for left ventricle segmentation. *Magnetic Resonance Imaging* (2020) 66:152–164. doi:](http://paperpile.com/b/T2eW7l/OtGie) [10.1016/j.mri.2019.08.004](http://dx.doi.org/10.1016/j.mri.2019.08.004)

93. [Lindsey T, Lee J-J. Automated Cardiovascular Pathology Assessment Using Semantic Segmentation and Ensemble Learning. *Journal of Digital Imaging* (2020) 33:607–612. doi:](http://paperpile.com/b/T2eW7l/VkqIF) [10.1007/s10278-019-00197-0](http://dx.doi.org/10.1007/s10278-019-00197-0)

94. [Liu J, Xie H, Zhang S, Gu L. Multi-sequence myocardium segmentation with cross-constrained shape and neural network-based initialization. *Computerized Medical Imaging and Graphics* (2019) 71:49–57. doi:](http://paperpile.com/b/T2eW7l/eK662) [10.1016/j.compmedimag.2018.11.001](http://dx.doi.org/10.1016/j.compmedimag.2018.11.001)

95. [Liu D, Jia Z, Jin M, Liu Q, Liao Z, Zhong J, Ye H, Chen G. Cardiac magnetic resonance image segmentation based on convolutional neural network. *Comput Methods Programs Biomed* (2020) 197:105755.](http://paperpile.com/b/T2eW7l/2N6Ip)

96. [Liu H, Hu H, Xu X, Song E. Automatic Left Ventricle Segmentation in Cardiac MRI Using Topological Stable-State Thresholding and Region Restricted Dynamic Programming. *Academic Radiology* (2012) 19:723–731. doi:](http://paperpile.com/b/T2eW7l/kR9oU) [10.1016/j.acra.2012.02.011](http://dx.doi.org/10.1016/j.acra.2012.02.011)

97. [Liu J, Zhuang X, Xie H, Zhang S, Gu L. Myocardium segmentation from DE MRI with guided random walks and sparse shape representation. *International Journal of Computer Assisted Radiology and Surgery* (2018) 13:1579–1590. doi:](http://paperpile.com/b/T2eW7l/9RNai) [10.1007/s11548-018-1817-4](http://dx.doi.org/10.1007/s11548-018-1817-4)

98. [Liu J, Zhuang X, Wu L, An D, Xu J, Peters T, Gu L. Myocardium Segmentation From DE MRI Using Multicomponent Gaussian Mixture Model and Coupled Level Set. *IEEE Trans Biomed Eng* (2017) 64:2650–2661.](http://paperpile.com/b/T2eW7l/zIaVI)

99. [Luo C, Shi C, Li X, Gao D. Cardiac MR segmentation based on sequence propagation by deep learning. *PLOS ONE* (2020) 15:e0230415. doi:](http://paperpile.com/b/T2eW7l/hUSqP) [10.1371/journal.pone.0230415](http://dx.doi.org/10.1371/journal.pone.0230415)

100. [Luo Y, Xu L, Qi L. A cascaded FC-DenseNet and level set method (FCDL) for fully automatic segmentation of the right ventricle in cardiac MRI. *Medical & Biological Engineering & Computing* (2021) 59:561–574. doi:](http://paperpile.com/b/T2eW7l/pvVxo) [10.1007/s11517-020-02305-7](http://dx.doi.org/10.1007/s11517-020-02305-7)

101. [Luo Y-G, Ko JKL, Shi L, Guan Y, Li L, Qin J, Heng P-A, Chu WCW, Wang D. Myocardial Iron Loading Assessment by Automatic Left Ventricle Segmentation with Morphological Operations and Geodesic Active Contour on T2* images. *Scientific Reports* (2015) 5: doi:](http://paperpile.com/b/T2eW7l/PmZ8L) [10.1038/srep12438](http://dx.doi.org/10.1038/srep12438)

102. [Luo G, Dong S, Wang W, Wang K, Cao S, Tam C, Zhang H, Howey J, Ohorodnyk P, Li S. Commensal correlation network between segmentation and direct area estimation for bi-ventricle quantification. *Medical Image Analysis* (2020) 59:101591. doi:](http://paperpile.com/b/T2eW7l/6qyiM) [10.1016/j.media.2019.101591](http://dx.doi.org/10.1016/j.media.2019.101591)

103. [Ma Z, Wu X, Wang X, Song Q, Yin Y, Cao K, Wang Y, Zhou J. An iterative multi‐path fully convolutional neural network for automatic cardiac segmentation in cine MR images. *Medical Physics* (2019) 46:5652–5665. doi:](http://paperpile.com/b/T2eW7l/R30M8) [10.1002/mp.13859](http://dx.doi.org/10.1002/mp.13859)

104. [Ma C, Luo G, Wang K. A Combined Random Forests and Active Contour Model Approach for Fully Automatic Segmentation of the Left Atrium in Volumetric MRI. *BioMed Research International* (2017) 2017:1–14. doi:](http://paperpile.com/b/T2eW7l/LIYqF) [10.1155/2017/8381094](http://dx.doi.org/10.1155/2017/8381094)

105. [Ma Y, Wang L, Ma Y, Dong M, Du S, Sun X. An SPCNN-GVF-based approach for the automatic segmentation of left ventricle in cardiac cine MR images. *International Journal of Computer Assisted Radiology and Surgery* (2016) 11:1951–1964. doi:](http://paperpile.com/b/T2eW7l/p2Ghs) [10.1007/s11548-016-1429-9](http://dx.doi.org/10.1007/s11548-016-1429-9)

106. [Ma T, Ma Z, Zhang X, Zhou F. Evaluation of Effect of Curcumin on Psychological State of Patients with Pulmonary Hypertension by Magnetic Resonance Image under Deep Learning. *Contrast Media Mol Imaging* (2021) 2021:9935754.](http://paperpile.com/b/T2eW7l/o06kk)

107. [Mahapatra D. Cardiac Image Segmentation from Cine Cardiac MRI Using Graph Cuts and Shape Priors. *Journal of Digital Imaging* (2013) 26:721–730. doi:](http://paperpile.com/b/T2eW7l/JWPVr) [10.1007/s10278-012-9548-5](http://dx.doi.org/10.1007/s10278-012-9548-5)

108. [Mahapatra D. Cardiac MRI Segmentation Using Mutual Context Information from Left and Right Ventricle. *Journal of Digital Imaging* (2013) 26:898–908. doi:](http://paperpile.com/b/T2eW7l/K20PW) [10.1007/s10278-013-9573-z](http://dx.doi.org/10.1007/s10278-013-9573-z)

109. [Mahapatra D. Automatic Cardiac Segmentation Using Semantic Information from Random Forests. *Journal of Digital Imaging* (2014) 27:794–804. doi:](http://paperpile.com/b/T2eW7l/uM6at) [10.1007/s10278-014-9705-0](http://dx.doi.org/10.1007/s10278-014-9705-0)

110. [Mamalakis M, Garg P, Nelson T, Lee J, Wild JM, Clayton RH. MA-SOCRATIS: An automatic pipeline for robust segmentation of the left ventricle and scar. *Computerized Medical Imaging and Graphics* (2021) 93:101982. doi:](http://paperpile.com/b/T2eW7l/Btr4y) [10.1016/j.compmedimag.2021.101982](http://dx.doi.org/10.1016/j.compmedimag.2021.101982)

111. [Matthew S, Gandy SJ, Nicholas RS, Waugh SA, Crowe EA, Lerski RA, Dunn MH, Houston JG. Quantitative analysis of cardiac left ventricular variables obtained by MRI at 3 T: a pre- and post-contrast comparison. *The British Journal of Radiology* (2012) 85:e343–e347. doi:](http://paperpile.com/b/T2eW7l/JEmzD) [10.1259/bjr/62891785](http://dx.doi.org/10.1259/bjr/62891785)

112. [Moccia S, Banali R, Martini C, Muscogiuri G, Pontone G, Pepi M, Caiani EG. Development and testing of a deep learning-based strategy for scar segmentation on CMR-LGE images. *Magnetic Resonance Materials in Physics, Biology and Medicine* (2019) 32:187–195. doi:](http://paperpile.com/b/T2eW7l/t7Q51) [10.1007/s10334-018-0718-4](http://dx.doi.org/10.1007/s10334-018-0718-4)

113. [Morais P, Queirós S, Heyde B, Engvall J, ’hooge JD, Vilaça JL. Fully automatic left ventricular myocardial strain estimation in 2D short-axis tagged magnetic resonance imaging. *Physics in Medicine & Biology* (2017) 62:6899–6919. doi:](http://paperpile.com/b/T2eW7l/mCnFk) [10.1088/1361-6560/aa7dc2](http://dx.doi.org/10.1088/1361-6560/aa7dc2)

114. [Morris ED, Ghanem AI, Pantelic MV, Walker EM, Han X, Glide-Hurst CK. Cardiac Substructure Segmentation and Dosimetry Using a Novel Hybrid Magnetic Resonance and Computed Tomography Cardiac Atlas. *Int J Radiat Oncol Biol Phys* (2019) 103:985–993.](http://paperpile.com/b/T2eW7l/yCytR)

115. [Nambakhsh CMS, Yuan J, Punithakumar K, Goela A, Rajchl M, Peters TM, Ayed IB. Left ventricle segmentation in MRI via convex relaxed distribution matching. *Med Image Anal* (2013) 17:1010–1024.](http://paperpile.com/b/T2eW7l/5OEWh)

116. [Ngo TA, Lu Z, Carneiro G. Combining deep learning and level set for the automated segmentation of the left ventricle of the heart from cardiac cine magnetic resonance. *Medical Image Analysis* (2017) 35:159–171. doi:](http://paperpile.com/b/T2eW7l/uXsCl) [10.1016/j.media.2016.05.009](http://dx.doi.org/10.1016/j.media.2016.05.009)

117. [Niu Y, Qin L, Wang X. Myocardium Detection by Deep SSAE Feature and Within-Class Neighborhood Preserved Support Vector Classifier and Regressor. *Sensors* (2019) 19:1766. doi:](http://paperpile.com/b/T2eW7l/8boTy) [10.3390/s19081766](http://dx.doi.org/10.3390/s19081766)

118. [Oktay O, Rueckert D, Bai W, Guerrero R, Rajchl M, de Marvao A, O’Regan DP, Cook SA, Heinrich MP, Glocker B. Stratified Decision Forests for Accurate Anatomical Landmark Localization in Cardiac Images. *IEEE Transactions on Medical Imaging* (2017) 36:332–342. doi:](http://paperpile.com/b/T2eW7l/aZ10J) [10.1109/tmi.2016.2597270](http://dx.doi.org/10.1109/tmi.2016.2597270)

119. [Paknezhad M, Marchesseau S, Brown MS. Automatic basal slice detection for cardiac analysis. *J Med Imaging (Bellingham)* (2016) 3:034004.](http://paperpile.com/b/T2eW7l/INPQB)

120. [Penso M, Moccia S, Scafuri S, Muscogiuri G, Pontone G, Pepi M, Caiani EG. Automated left and right ventricular chamber segmentation in cardiac magnetic resonance images using dense fully convolutional neural network. *Comput Methods Programs Biomed* (2021) 204:106059.](http://paperpile.com/b/T2eW7l/TnJbJ)

121. [Pérez-Pelegrí M, Monmeneu JV, López-Lereu MP, Pérez-Pelegrí L, Maceira AM, Bodí V, Moratal D. Automatic left ventricle volume calculation with explainability through a deep learning weak-supervision methodology. *Comput Methods Programs Biomed* (2021) 208:106275.](http://paperpile.com/b/T2eW7l/rcmrB)

122. [Punithakumar K, Ben Ayed I, Islam A, Goela A, Ross IG, Chong J, Li S. Regional heart motion abnormality detection: an information theoretic approach. *Med Image Anal* (2013) 17:311–324.](http://paperpile.com/b/T2eW7l/Heuqv)

123. [Punithakumar K, Noga M, Ben Ayed I, Boulanger P. Right ventricular segmentation in cardiac MRI with moving mesh correspondences. *Comput Med Imaging Graph* (2015) 43:15–25.](http://paperpile.com/b/T2eW7l/eryhC)

124. [Puyol-Antón E, Ruijsink B, Baumgartner CF, Masci P-G, Sinclair M, Konukoglu E, Razavi R, King AP. Automated quantification of myocardial tissue characteristics from native T1 mapping using neural networks with uncertainty-based quality-control. *J Cardiovasc Magn Reson* (2020) 22:60.](http://paperpile.com/b/T2eW7l/ZwlYb)

125. [Qin W, Wu Y, Li S, Chen Y, Yang Y, Liu X, Zheng H, Liang D, Hu Z. Automated segmentation of the left ventricle from MR cine imaging based on deep learning architecture. *Biomed Phys Eng Express* (2020) 6:025009.](http://paperpile.com/b/T2eW7l/fxyry)

126. [Queirós S, Vilaça JL, Morais P, Fonseca JC, D’hooge J, Barbosa D. Fast left ventricle tracking using localized anatomical affine optical flow. *International Journal for Numerical Methods in Biomedical Engineering* (2017) 33: doi:](http://paperpile.com/b/T2eW7l/RR5BQ) [10.1002/cnm.2871](http://dx.doi.org/10.1002/cnm.2871)

127. [Queirós S, Barbosa D, Heyde B, Morais P, Vilaça JL, Friboulet D, Bernard O, D’hooge J. Fast automatic myocardial segmentation in 4D cine CMR datasets. *Medical Image Analysis* (2014) 18:1115–1131. doi:](http://paperpile.com/b/T2eW7l/DFUL9) [10.1016/j.media.2014.06.001](http://dx.doi.org/10.1016/j.media.2014.06.001)

128. [Queirós S, Barbosa D, Engvall J, Ebbers T, Nagel E, Sarvari SI, Claus P, Fonseca JC, Vilaça JL, D’hooge J. Multi-centre validation of an automatic algorithm for fast 4D myocardial segmentation in cine CMR datasets. *European Heart Journal – Cardiovascular Imaging* (2016) 17:1118–1127. doi:](http://paperpile.com/b/T2eW7l/DlDxy) [10.1093/ehjci/jev247](http://dx.doi.org/10.1093/ehjci/jev247)

129. [Razeghi O, Sim I, Roney CH, Karim R, Chubb H, Whitaker J, O’Neill L, Mukherjee R, Wright M, O’Neill M, et al. Fully Automatic Atrial Fibrosis Assessment Using a Multilabel Convolutional Neural Network. *Circulation: Cardiovascular Imaging* (2020) 13: doi:](http://paperpile.com/b/T2eW7l/TfzxN) [10.1161/circimaging.120.011512](http://dx.doi.org/10.1161/circimaging.120.011512)

130. [Ringenberg J, Deo M, Devabhaktuni V, Berenfeld O, Boyers P, Gold J. Fast, accurate, and fully automatic segmentation of the right ventricle in short-axis cardiac MRI. *Computerized Medical Imaging and Graphics* (2014) 38:190–201. doi:](http://paperpile.com/b/T2eW7l/4cKM2) [10.1016/j.compmedimag.2013.12.011](http://dx.doi.org/10.1016/j.compmedimag.2013.12.011)

131. [Romaguera LV, Romero FP, Fernandes Costa Filho CF, Fernandes Costa MG. Myocardial segmentation in cardiac magnetic resonance images using fully convolutional neural networks. *Biomed Signal Process Control* (2018) 44:48–57.](http://paperpile.com/b/T2eW7l/fWzfS)

132. [Rostami A, Amirani MC, Yousef-Banaem H. Segmentation of the left ventricle in cardiac MRI based on convolutional neural network and level set function. *Health and Technology* (2020) 10:1155–1162. doi:](http://paperpile.com/b/T2eW7l/LC2lA) [10.1007/s12553-020-00461-2](http://dx.doi.org/10.1007/s12553-020-00461-2)

133. [Ruijsink B, Puyol-Antón E, Oksuz I, Sinclair M, Bai W, Schnabel JA, Razavi R, King AP. Fully Automated, Quality-Controlled Cardiac Analysis From CMR: Validation and Large-Scale Application to Characterize Cardiac Function. *JACC Cardiovasc Imaging* (2020) 13:684–695.](http://paperpile.com/b/T2eW7l/fPGbw)

134. [Sander J, de Vos BD, Išgum I. Automatic segmentation with detection of local segmentation failures in cardiac MRI. *Sci Rep* (2020) 10:21769.](http://paperpile.com/b/T2eW7l/0ohoj)

135. [Sandfort V, Jacobs M, Arai AE, Hsu L-Y. Reliable segmentation of 2D cardiac magnetic resonance perfusion image sequences using time as the 3rd dimension. *European Radiology* (2021) 31:3941–3950. doi:](http://paperpile.com/b/T2eW7l/A7rRN) [10.1007/s00330-020-07474-5](http://dx.doi.org/10.1007/s00330-020-07474-5)

136. [Scannell CM, Veta M, Villa ADM, Sammut EC, Lee J, Breeuwer M, Chiribiri A. Deep-Learning-Based Preprocessing for Quantitative Myocardial Perfusion MRI. *J Magn Reson Imaging* (2020) 51:1689–1696.](http://paperpile.com/b/T2eW7l/54Yh2)

137. [Shaaf ZF, Jamil MMA, Ambar R, Alattab AA, Yahya AA, Asiri Y. Automatic Left Ventricle Segmentation from Short-Axis Cardiac MRI Images Based on Fully Convolutional Neural Network. *Diagnostics (Basel)* (2022) 12: doi:](http://paperpile.com/b/T2eW7l/7aM9k) [10.3390/diagnostics12020414](http://dx.doi.org/10.3390/diagnostics12020414)

138. [Shahzad R, Tao Q, Dzyubachyk O, Staring M, Lelieveldt BPF, van der Geest RJ. Fully-automatic left ventricular segmentation from long-axis cardiac cine MR scans. *Medical Image Analysis* (2017) 39:44–55. doi:](http://paperpile.com/b/T2eW7l/IY03e) [10.1016/j.media.2017.04.004](http://dx.doi.org/10.1016/j.media.2017.04.004)

139. [Sharma K, Alsadoon A, Prasad PWC, Al-Dala’in T, Nguyen TQV, Pham DTH. A novel solution of using deep learning for left ventricle detection: Enhanced feature extraction. *Computer Methods and Programs in Biomedicine* (2020) 197:105751. doi:](http://paperpile.com/b/T2eW7l/UooZ0) [10.1016/j.cmpb.2020.105751](http://dx.doi.org/10.1016/j.cmpb.2020.105751)

140. [Shen D, Pathrose A, Sarnari R, Blake A, Berhane H, Baraboo JJ, Carr JC, Markl M, Kim D. Automated segmentation of biventricular contours in tissue phase mapping using deep learning. *NMR Biomed* (2021) 34:e4606.](http://paperpile.com/b/T2eW7l/P9tHq)

141. [Shi J, Ye Y, Zhu D, Su L, Huang Y, Huang J. Automatic segmentation of cardiac magnetic resonance images based on multi-input fusion network. *Computer Methods and Programs in Biomedicine* (2021) 209:106323. doi:](http://paperpile.com/b/T2eW7l/jZvOm) [10.1016/j.cmpb.2021.106323](http://dx.doi.org/10.1016/j.cmpb.2021.106323)

142. [Shi X, Li C. Convexity preserving level set for left ventricle segmentation. *Magnetic Resonance Imaging* (2021) 78:109–118. doi:](http://paperpile.com/b/T2eW7l/mT4cC) [10.1016/j.mri.2021.02.003](http://dx.doi.org/10.1016/j.mri.2021.02.003)

143. [Simantiris G, Tziritas G. Cardiac MRI Segmentation With a Dilated CNN Incorporating Domain-Specific Constraints. *IEEE Journal of Selected Topics in Signal Processing* (2020) 14:1235–1243. doi:](http://paperpile.com/b/T2eW7l/AZJFL) [10.1109/jstsp.2020.3013351](http://dx.doi.org/10.1109/jstsp.2020.3013351)

144. [Sliman H, Khalifa F, Elnakib A, Soliman A, El-Baz A, Beache GM, Elmaghraby A, Gimel’farb G. Myocardial borders segmentation from cine MR images using bidirectional coupled parametric deformable models. *Medical Physics* (2013) 40:092302. doi:](http://paperpile.com/b/T2eW7l/HUBV8) [10.1118/1.4817478](http://dx.doi.org/10.1118/1.4817478)

145. [Suinesiaputra A, Cowan BR, Al-Agamy AO, Elattar MA, Ayache N, Fahmy AS, Khalifa AM, Medrano-Gracia P, Jolly M-P, Kadish AH, et al. A collaborative resource to build consensus for automated left ventricular segmentation of cardiac MR images. *Med Image Anal* (2014) 18:50–62.](http://paperpile.com/b/T2eW7l/lAFfG)

146. [Suinesiaputra A, Sanghvi MM, Aung N, Paiva JM, Zemrak F, Fung K, Lukaschuk E, Lee AM, Carapella V, Kim YJ, et al. Fully-automated left ventricular mass and volume MRI analysis in the UK Biobank population cohort: evaluation of initial results. *The International Journal of Cardiovascular Imaging* (2018) 34:281–291. doi:](http://paperpile.com/b/T2eW7l/bm6ur) [10.1007/s10554-017-1225-9](http://dx.doi.org/10.1007/s10554-017-1225-9)

147. [Sun X, Garg P, Plein S, van der Geest RJ. SAUN: Stack attention U-Net for left ventricle segmentation from cardiac cine magnetic resonance imaging. *Med Phys* (2021) 48:1750–1763.](http://paperpile.com/b/T2eW7l/2EJTn)

148. [Tan LK, Liew YM, Lim E, McLaughlin RA. Convolutional neural network regression for short-axis left ventricle segmentation in cardiac cine MR sequences. *Med Image Anal* (2017) 39:78–86.](http://paperpile.com/b/T2eW7l/qoTj1)

149. [Tan LK, McLaughlin RA, Lim E, Aziz YFA, Liew YM. Fully automated segmentation of the left ventricle in cine cardiac MRI using neural network regression. *Journal of Magnetic Resonance Imaging* (2018) 48:140–152. doi:](http://paperpile.com/b/T2eW7l/fwx9q) [10.1002/jmri.25932](http://dx.doi.org/10.1002/jmri.25932)

150. [Tan LK, Liew YM, Lim E, Aziz YFA, Chee KH, McLaughlin RA. Automatic localization of the left ventricular blood pool centroid in short axis cardiac cine MR images. *Medical & Biological Engineering & Computing* (2018) 56:1053–1062. doi:](http://paperpile.com/b/T2eW7l/6zN0v) [10.1007/s11517-017-1750-7](http://dx.doi.org/10.1007/s11517-017-1750-7)

151. [Tandon A, Mohan N, Jensen C, Burkhardt BEU, Gooty V, Castellanos DA, McKenzie PL, Zahr RA, Bhattaru A, Abdulkarim M, et al. Retraining Convolutional Neural Networks for Specialized Cardiovascular Imaging Tasks: Lessons from Tetralogy of Fallot. *Pediatr Cardiol* (2021) 42:578–589.](http://paperpile.com/b/T2eW7l/WxShg)

152. [Tao Q, Yan W, Wang Y, Paiman EHM, Shamonin DP, Garg P, Plein S, Huang L, Xia L, Sramko M, et al. Deep Learning–based Method for Fully Automatic Quantification of Left Ventricle Function from Cine MR Images: A Multivendor, Multicenter Study. *Radiology* (2019) 290:81–88.](http://paperpile.com/b/T2eW7l/OLp6M)

153. [Tao Q, Piers SRD, Lamb HJ, van der Geest RJ. Automated left ventricle segmentation in late gadolinium-enhanced MRI for objective myocardial scar assessment. *Journal of Magnetic Resonance Imaging* (2015) 42:390–399. doi:](http://paperpile.com/b/T2eW7l/W10HA) [10.1002/jmri.24804](http://dx.doi.org/10.1002/jmri.24804)

154. [Tao Q, Ipek EG, Shahzad R, Berendsen FF, Nazarian S, van der Geest RJ. Fully automatic segmentation of left atrium and pulmonary veins in late gadolinium-enhanced MRI: Towards objective atrial scar assessment. *J Magn Reson Imaging* (2016) 44:346–354.](http://paperpile.com/b/T2eW7l/Ma1oN)

155. [Tarroni G, Oktay O, Bai W, Schuh A, Suzuki H, Passerat-Palmbach J, de Marvao A, O’Regan DP, Cook S, Glocker B, et al. Learning-Based Quality Control for Cardiac MR Images. *IEEE Trans Med Imaging* (2019) 38:1127–1138.](http://paperpile.com/b/T2eW7l/cpqFr)

156. [Tobon-Gomez C, Sukno FM, Butakoff C, Huguet M, Frangi AF. Automatic training and reliability estimation for 3D ASM applied to cardiac MRI segmentation. *Physics in Medicine and Biology* (2012) 57:4155–4174. doi:](http://paperpile.com/b/T2eW7l/9HYMj) [10.1088/0031-9155/57/13/4155](http://dx.doi.org/10.1088/0031-9155/57/13/4155)

157. [Tong Q, Li C, Si W, Liao X, Tong Y, Yuan Z, Heng PA. RIANet: Recurrent interleaved attention network for cardiac MRI segmentation. *Computers in Biology and Medicine* (2019) 109:290–302. doi:](http://paperpile.com/b/T2eW7l/zf7dU) [10.1016/j.compbiomed.2019.04.042](http://dx.doi.org/10.1016/j.compbiomed.2019.04.042)

158. [Tsadok Y, Petrank Y, Sarvari S, Edvardsen T, Adam D. Automatic segmentation of cardiac MRI cines validated for long axis views. *Computerized Medical Imaging and Graphics* (2013) 37:500–511. doi:](http://paperpile.com/b/T2eW7l/wNX2R) [10.1016/j.compmedimag.2013.09.002](http://dx.doi.org/10.1016/j.compmedimag.2013.09.002)

159. [Tufvesson J, Carlsson M, Aletras AH, Engblom H, Deux J-F, Koul S, Sörensson P, Pernow J, Atar D, Erlinge D, et al. Automatic segmentation of myocardium at risk from contrast enhanced SSFP CMR: validation against expert readers and SPECT. *BMC Medical Imaging* (2016) 16: doi:](http://paperpile.com/b/T2eW7l/iUniC) [10.1186/s12880-016-0124-1](http://dx.doi.org/10.1186/s12880-016-0124-1)

160. [Ukwatta E, Yuan J, Qiu W, Wu KC, Trayanova N, Vadakkumpadan F. Myocardial Infarct Segmentation and Reconstruction from 2D Late-Gadolinium Enhanced Magnetic Resonance Images. *Medical Image Computing and Computer-Assisted Intervention – MICCAI 2014* (2014)554–561. doi:](http://paperpile.com/b/T2eW7l/f88gl) [10.1007/978-3-319-10470-6_69](http://dx.doi.org/10.1007/978-3-319-10470-6_69)

161. [Valinoti M, Fabbri C, Turco D, Mantovan R, Pasini A, Corsi C. 3D patient-specific models for left atrium characterization to support ablation in atrial fibrillation patients. *Magnetic Resonance Imaging* (2018) 45:51–57. doi:](http://paperpile.com/b/T2eW7l/EC7Uf) [10.1016/j.mri.2017.09.012](http://dx.doi.org/10.1016/j.mri.2017.09.012)

162. [Veni G, Fu Z, Awate SP, Whitaker RT. Bayesian segmentation of atrium wall using globally-optimal graph cuts on 3D meshes. *Inf Process Med Imaging* (2013) 23:656–667.](http://paperpile.com/b/T2eW7l/pbQWv)

163. [Vesal S, Gu M, Maier A, Ravikumar N. Spatio-Temporal Multi-Task Learning for Cardiac MRI Left Ventricle Quantification. *IEEE J Biomed Health Inform* (2021) 25:2698–2709.](http://paperpile.com/b/T2eW7l/LkGoP)

164. [Vigneault DM, Xie W, Ho CY, Bluemke DA, Noble JA. Ω-Net (Omega-Net): Fully automatic, multi-view cardiac MR detection, orientation, and segmentation with deep neural networks. *Med Image Anal* (2018) 48:95–106.](http://paperpile.com/b/T2eW7l/w7D5k)

165. [Wang Z, Xie L, Qi J. Dynamic pixel-wise weighting-based fully convolutional neural networks for left ventricle segmentation in short-axis MRI. *Magn Reson Imaging* (2020) 66:131–140.](http://paperpile.com/b/T2eW7l/zfD6a)

166. [Wang T, Wang J, Zhao J, Zhang Y. A Myocardial Segmentation Method Based on Adversarial Learning. *Biomed Res Int* (2021) 2021:6618918.](http://paperpile.com/b/T2eW7l/7EBM6)

167. [Wang Y, Zhang Y, Wen Z, Tian B, Kao E, Liu X, Xuan W, Ordovas K, Saloner D, Liu J. Deep learning based fully automatic segmentation of the left ventricular endocardium and epicardium from cardiac cine MRI. *Quant Imaging Med Surg* (2021) 11:1600–1612.](http://paperpile.com/b/T2eW7l/s5IjU)

168. [Wang L, Pei M, Codella NCF, Kochar M, Weinsaft JW, Li J, Prince MR, Wang Y. Left Ventricle: Fully Automated Segmentation Based on Spatiotemporal Continuity and Myocardium Information in Cine Cardiac Magnetic Resonance Imaging (LV-FAST). *BioMed Research International* (2015) 2015:1–9. doi:](http://paperpile.com/b/T2eW7l/tWoJK) [10.1155/2015/367583](http://dx.doi.org/10.1155/2015/367583)

169. [Wang Y, Zhang Y, Xuan W, Kao E, Cao P, Tian B, Ordovas K, Saloner D, Liu J. Fully automatic segmentation of 4D MRI for cardiac functional measurements. *Medical Physics* (2019) 46:180–189. doi:](http://paperpile.com/b/T2eW7l/IYkMk) [10.1002/mp.13245](http://dx.doi.org/10.1002/mp.13245)

170. [Wang B, Gu X, Fan C, Xie H, Zhang S, Tian X, Gu L. Sparse group composition for robust left ventricular epicardium segmentation. *Computerized Medical Imaging and Graphics* (2015) 46:56–63. doi:](http://paperpile.com/b/T2eW7l/HX5IC) [10.1016/j.compmedimag.2015.06.003](http://dx.doi.org/10.1016/j.compmedimag.2015.06.003)

171. [Wang X, Zhai S, Niu Y. Left ventricle landmark localization and identification in cardiac MRI by deep metric learning-assisted CNN regression. *Neurocomputing* (2020) 399:153–170. doi:](http://paperpile.com/b/T2eW7l/RDE8o) [10.1016/j.neucom.2020.02.069](http://dx.doi.org/10.1016/j.neucom.2020.02.069)

172. [Wantanajittikul K, Theera-Umpon N, Saekho S, Auephanwiriyakul S, Phrommintikul A, Leemasawat K. Automatic cardiac T2* relaxation time estimation from magnetic resonance images using region growing method with automatically initialized seed points. *Comput Methods Programs Biomed* (2016) 130:76–86.](http://paperpile.com/b/T2eW7l/92SLT)

173. [Wech T, Ankenbrand MJ, Bley TA, Heidenreich JF. A data-driven semantic segmentation model for direct cardiac functional analysis based on undersampled radial MR cine series. *Magn Reson Med* (2022) 87:972–983.](http://paperpile.com/b/T2eW7l/FjAcR)

174. [Wei D, Sun Y, Ong S-H, Chai P, Teo LL, Low AF. A comprehensive 3-D framework for automatic quantification of late gadolinium enhanced cardiac magnetic resonance images. *IEEE Trans Biomed Eng* (2013) 60:1499–1508.](http://paperpile.com/b/T2eW7l/hezcI)

175. [Wei D, Sun Y, Ong S-H, Chai P, Teo LL, Low AF. Three-dimensional segmentation of the left ventricle in late gadolinium enhanced MR images of chronic infarction combining long- and short-axis information. *Medical Image Analysis* (2013) 17:685–697. doi:](http://paperpile.com/b/T2eW7l/sGF2H) [10.1016/j.media.2013.03.001](http://dx.doi.org/10.1016/j.media.2013.03.001)

176. [Woie L, Engan K, Eftestøl T, Larsen AI, Ørn S. The Localization and Characterization of Ischemic Scars in relation to the Infarct Related Coronary Artery Assessed by Cardiac Magnetic Resonance and a Novel Automatic Postprocessing Method. *Cardiology Research and Practice* (2015) 2015:1–9. doi:](http://paperpile.com/b/T2eW7l/ULoTr) [10.1155/2015/120874](http://dx.doi.org/10.1155/2015/120874)

177. [Wu H, Lu X, Lei B, Wen Z. Automated left ventricular segmentation from cardiac magnetic resonance images via adversarial learning with multi-stage pose estimation network and co-discriminator. *Medical Image Analysis* (2021) 68:101891. doi:](http://paperpile.com/b/T2eW7l/TsfjR) [10.1016/j.media.2020.101891](http://dx.doi.org/10.1016/j.media.2020.101891)

178. [Wu Z-H, Sun L-P, Liu Y-L, Dong D-D, Tong L, Deng D-D, He Y, Wang H, Sun Y-B, Dong J-Z, et al. Fully Automatic Scar Segmentation for Late Gadolinium Enhancement MRI Images in Left Ventricle with Myocardial Infarction. *Curr Med Sci* (2021) 41:398–404.](http://paperpile.com/b/T2eW7l/rGw9t)

179. [Wu B, Fang Y, Lai X. Left ventricle automatic segmentation in cardiac MRI using a combined CNN and U-net approach. *Computerized Medical Imaging and Graphics* (2020) 82:101719. doi:](http://paperpile.com/b/T2eW7l/s9aPB) [10.1016/j.compmedimag.2020.101719](http://dx.doi.org/10.1016/j.compmedimag.2020.101719)

180. [Wu Y, Hatipoglu S, Alonso-Álvarez D, Gatehouse P, Li B, Gao Y, Firmin D, Keegan J, Yang G. Fast and Automated Segmentation for the Three-Directional Multi-Slice Cine Myocardial Velocity Mapping. *Diagnostics (Basel)* (2021) 11: doi:](http://paperpile.com/b/T2eW7l/B91rw) [10.3390/diagnostics11020346](http://dx.doi.org/10.3390/diagnostics11020346)

181. [Wu J, Mazur TR, Ruan S, Lian C, Daniel N, Lashmett H, Ochoa L, Zoberi I, Anastasio MA, Michael Gach H, et al. A deep Boltzmann machine-driven level set method for heart motion tracking using cine MRI images. *Medical Image Analysis* (2018) 47:68–80. doi:](http://paperpile.com/b/T2eW7l/V9g1J) [10.1016/j.media.2018.03.015](http://dx.doi.org/10.1016/j.media.2018.03.015)

182. [Wu J, Gan Z, Guo W, Yang X, Lin A. A fully convolutional network feature descriptor: Application to left ventricle motion estimation based on graph matching in short-axis MRI. *Neurocomputing* (2020) 392:196–208. doi:](http://paperpile.com/b/T2eW7l/ssvLY) [10.1016/j.neucom.2018.10.101](http://dx.doi.org/10.1016/j.neucom.2018.10.101)

183. [Xie L, Song Y, Chen Q. Automatic left ventricle segmentation in short-axis MRI using deep convolutional neural networks and central-line guided level set approach. *Comput Biol Med* (2020) 122:103877.](http://paperpile.com/b/T2eW7l/L7rgN)

184. [Xiong Z, Fedorov VV, Fu X, Cheng E, Macleod R, Zhao J. Fully Automatic Left Atrium Segmentation From Late Gadolinium Enhanced Magnetic Resonance Imaging Using a Dual Fully Convolutional Neural Network. *IEEE Trans Med Imaging* (2019) 38:515–524.](http://paperpile.com/b/T2eW7l/E3FVC)

185. [Xu C, Howey J, Ohorodnyk P, Roth M, Zhang H, Li S. Segmentation and quantification of infarction without contrast agents via spatiotemporal generative adversarial learning. *Medical Image Analysis* (2020) 59:101568. doi:](http://paperpile.com/b/T2eW7l/YMSjR) [10.1016/j.media.2019.101568](http://dx.doi.org/10.1016/j.media.2019.101568)

186. [Xu C, Xu L, Ohorodnyk P, Roth M, Chen B, Li S. Contrast agent-free synthesis and segmentation of ischemic heart disease images using progressive sequential causal GANs. *Med Image Anal* (2020) 62:101668.](http://paperpile.com/b/T2eW7l/DrTNi)

187. [Xu C, Xu L, Gao Z, Zhao S, Zhang H, Zhang Y, Du X, Zhao S, Ghista D, Liu H, et al. Direct delineation of myocardial infarction without contrast agents using a joint motion feature learning architecture. *Med Image Anal* (2018) 50:82–94.](http://paperpile.com/b/T2eW7l/zl94c)

188. [Xue H, Davies RH, Brown LAE, Knott KD, Kotecha T, Fontana M, Plein S, Moon JC, Kellman P. Automated Inline Analysis of Myocardial Perfusion MRI with Deep Learning. *Radiol Artif Intell* (2020) 2:e200009.](http://paperpile.com/b/T2eW7l/3V040)

189. [Xue H, Tseng E, Knott KD, Kotecha T, Brown L, Plein S, Fontana M, Moon JC, Kellman P. Automated detection of left ventricle in arterial input function images for inline perfusion mapping using deep learning: A study of 15,000 patients. *Magnetic Resonance in Medicine* (2020) 84:2788–2800. doi:](http://paperpile.com/b/T2eW7l/RrljT) [10.1002/mrm.28291](http://dx.doi.org/10.1002/mrm.28291)

190. [Yan W, Wang Y, van der Geest RJ, Tao Q. Cine MRI analysis by deep learning of optical flow: Adding the temporal dimension. *Computers in Biology and Medicine* (2019) 111:103356. doi:](http://paperpile.com/b/T2eW7l/xYOHv) [10.1016/j.compbiomed.2019.103356](http://dx.doi.org/10.1016/j.compbiomed.2019.103356)

191. [Yan W, Huang L, Xia L, Gu S, Yan F, Wang Y, Tao Q. MRI Manufacturer Shift and Adaptation: Increasing the Generalizability of Deep Learning Segmentation for MR Images Acquired with Different Scanners. *Radiol Artif Intell* (2020) 2:e190195.](http://paperpile.com/b/T2eW7l/m7WJW)

192. [Yang G, Zhuang X, Khan H, Haldar S, Nyktari E, Li L, Wage R, Ye X, Slabaugh G, Mohiaddin R, et al. Fully automatic segmentation and objective assessment of atrial scars for long-standing persistent atrial fibrillation patients using late gadolinium-enhanced MRI. *Medical Physics* (2018) 45:1562–1576. doi:](http://paperpile.com/b/T2eW7l/wcy6z) [10.1002/mp.12832](http://dx.doi.org/10.1002/mp.12832)

193. [Yang F, Zhang Y, Lei P, Wang L, Miao Y, Xie H, Zeng Z. A Deep Learning Segmentation Approach in Free-Breathing Real-Time Cardiac Magnetic Resonance Imaging. *Biomed Res Int* (2019) 2019:5636423.](http://paperpile.com/b/T2eW7l/khRIz)

194. [Yang X, Zhang Y, Lo B, Wu D, Liao H, Zhang Y-T. DBAN: Adversarial Network With Multi-Scale Features for Cardiac MRI Segmentation. *IEEE J Biomed Health Inform* (2021) 25:2018–2028.](http://paperpile.com/b/T2eW7l/S0yCd)

195. [Yang X, Song Q, Su Y. Automatic segmentation of left ventricle cavity from short-axis cardiac magnetic resonance images. *Med Biol Eng Comput* (2017) 55:1563–1577.](http://paperpile.com/b/T2eW7l/WZuoU)

196. [Zabihollahy F, White JA, Ukwatta E. Convolutional neural network-based approach for segmentation of left ventricle myocardial scar from 3D late gadolinium enhancement MR images. *Med Phys* (2019) 46:1740–1751.](http://paperpile.com/b/T2eW7l/xMHdc)

197. [Zabihollahy F, Rajchl M, White JA, Ukwatta E. Fully automated segmentation of left ventricular scar from 3D late gadolinium enhancement magnetic resonance imaging using a cascaded multi-planar U-Net (CMPU-Net). *Med Phys* (2020) 47:1645–1655.](http://paperpile.com/b/T2eW7l/Y9PDA)

198. [Zarvani M, Saberi S, Azmi R, Shojaedini SV. Residual Learning: A New Paradigm to Improve Deep Learning-Based Segmentation of the Left Ventricle in Magnetic Resonance Imaging Cardiac Images. *J Med Signals Sens* (2021) 11:159–168.](http://paperpile.com/b/T2eW7l/QIq4C)

199. [Zhang H, Zhang W, Shen W, Li N, Chen Y, Li S, Chen B, Guo S, Wang Y. Automatic segmentation of the cardiac MR images based on nested fully convolutional dense network with dilated convolution. *Biomed Signal Process Control* (2021) 68:102684.](http://paperpile.com/b/T2eW7l/a6irF)

200. [Zhang X, Noga M, Martin DG, Punithakumar K. Fully automated left atrium segmentation from anatomical cine long-axis MRI sequences using deep convolutional neural network with unscented Kalman filter. *Medical Image Analysis* (2021) 68:101916. doi:](http://paperpile.com/b/T2eW7l/JavOz) [10.1016/j.media.2020.101916](http://dx.doi.org/10.1016/j.media.2020.101916)

201. [Zhang N, Yang G, Gao Z, Xu C, Zhang Y, Shi R, Keegan J, Xu L, Zhang H, Fan Z, et al. Deep Learning for Diagnosis of Chronic Myocardial Infarction on Nonenhanced Cardiac Cine MRI. *Radiology* (2019) 291:606–617. doi:](http://paperpile.com/b/T2eW7l/us9VJ) [10.1148/radiol.2019182304](http://dx.doi.org/10.1148/radiol.2019182304)

202. [Zhao M, Wei Y, Lu Y, Wong KKL. A novel U-Net approach to segment the cardiac chamber in magnetic resonance images with ghost artifacts. *Comput Methods Programs Biomed* (2020) 196:105623.](http://paperpile.com/b/T2eW7l/tk1cX)

203. [Zheng Q, Delingette H, Duchateau N, Ayache N. 3-D Consistent and Robust Segmentation of Cardiac Images by Deep Learning With Spatial Propagation. *IEEE Trans Med Imaging* (2018) 37:2137–2148.](http://paperpile.com/b/T2eW7l/7H1dS)

204. [Zheng Q, Feng Y, Wei X, Feng M, Chen W, Lu Z, Xu Y, Chen H, He T. Automated interventricular septum segmentation for black-blood myocardial T2* measurement in thalassemia. *Journal of Magnetic Resonance Imaging* (2015) 41:1242–1250. doi:](http://paperpile.com/b/T2eW7l/6TSxT) [10.1002/jmri.24662](http://dx.doi.org/10.1002/jmri.24662)

205. [Zheng Q, Lu Z, Zhang M, Xu L, Ma H, Song S, Feng Q, Feng Y, Chen W, He T. Automatic Segmentation of Myocardium from Black-Blood MR Images Using Entropy and Local Neighborhood Information. *PLOS ONE* (2015) 10:e0120018. doi:](http://paperpile.com/b/T2eW7l/UvzcI) [10.1371/journal.pone.0120018](http://dx.doi.org/10.1371/journal.pone.0120018)

206. [Zhu Y, Fahmy AS, Duan C, Nakamori S, Nezafat R. Automated Myocardial T2 and Extracellular Volume Quantification in Cardiac MRI Using Transfer Learning–based Myocardium Segmentation. *Radiology: Artificial Intelligence* (2020) 2:e190034. doi:](http://paperpile.com/b/T2eW7l/9IMNw) [10.1148/ryai.2019190034](http://dx.doi.org/10.1148/ryai.2019190034)

207. [Zhu L, Gao Y, Yezzi A, Tannenbaum A. Automatic Segmentation of the Left Atrium From MR Images via Variational Region Growing With a Moments-Based Shape Prior. *IEEE Transactions on Image Processing* (2013) 22:5111–5122. doi:](http://paperpile.com/b/T2eW7l/9HAEj) [10.1109/tip.2013.2282049](http://dx.doi.org/10.1109/tip.2013.2282049)

208. [Zotti C, Luo Z, Lalande A, Jodoin P-M. Convolutional Neural Network With Shape Prior Applied to Cardiac MRI Segmentation. *IEEE J Biomed Health Inform* (2019) 23:1119–1128.](http://paperpile.com/b/T2eW7l/Z8uaf)

209. [Mongan J, Moy L, Kahn CE Jr. Checklist for Artificial Intelligence in Medical Imaging (CLAIM): A Guide for Authors and Reviewers. *Radiol Artif Intell* (2020) 2:e200029.](http://paperpile.com/b/T2eW7l/6RTV)

210. [Radau P, Lu Y, Connelly K, Paul G, Dick A, Wright G. Evaluation framework for algorithms segmenting short axis cardiac MRI. *The MIDAS Journal-Cardiac MR Left Ventricle Segmentation Challenge* (2009) 49:](http://paperpile.com/b/T2eW7l/rVUaE)

211. [Karim R, Bhagirath P, Claus P, James Housden R, Chen Z, Karimaghaloo Z, Sohn H-M, Lara Rodríguez L, Vera S, Albà X, et al. Evaluation of state-of-the-art segmentation algorithms for left ventricle infarct from late Gadolinium enhancement MR images. *Med Image Anal* (2016) 30:95–107.](http://paperpile.com/b/T2eW7l/kWUoL)

212. [Petitjean C, Zuluaga MA, Bai W, Dacher J-N, Grosgeorge D, Caudron J, Ruan S, Ayed IB, Cardoso MJ, Chen H-C, et al. Right ventricle segmentation from cardiac MRI: a collation study. *Med Image Anal* (2015) 19:187–202.](http://paperpile.com/b/T2eW7l/X1jzb)

213. [Asman A, Akhondi-Asl A, Wang H, Tustison N, Avants B, Warfield SK, Landman B. Miccai 2013 segmentation algorithms, theory and applications (SATA) challenge results summary. *MICCAI Challenge Workshop on Segmentation: Algorithms, Theory and Applications (SATA)*. (2013)](http://paperpile.com/b/T2eW7l/j6dpf)

214. [Pace DF, Dalca AV, Geva T, Powell AJ, Moghari MH, Golland P. Interactive Whole-Heart Segmentation in Congenital Heart Disease. *Med Image Comput Comput Assist Interv* (2015) 9351:80–88.](http://paperpile.com/b/T2eW7l/NUPGh)

215. [Bernard O, Lalande A, Zotti C, Cervenansky F, Yang X, Heng P-A, Cetin I, Lekadir K, Camara O, Gonzalez Ballester MA, et al. Deep Learning Techniques for Automatic MRI Cardiac Multi-Structures Segmentation and Diagnosis: Is the Problem Solved? *IEEE Trans Med Imaging* (2018) 37:2514–2525.](http://paperpile.com/b/T2eW7l/jIfHg)

216. [Zhuang X, Shen J. Multi-scale patch and multi-modality atlases for whole heart segmentation of MRI. *Med Image Anal* (2016) 31:77–87.](http://paperpile.com/b/T2eW7l/fjdxy)

217. [Xiong Z, Xia Q, Hu Z, Huang N, Bian C, Zheng Y, Vesal S, Ravikumar N, Maier A, Yang X, et al. A global benchmark of algorithms for segmenting the left atrium from late gadolinium-enhanced cardiac magnetic resonance imaging. *Med Image Anal* (2021) 67:101832.](http://paperpile.com/b/T2eW7l/EWRc2)

218. [Xue W, Li J, Hu Z, Kerfoot E, Clough J, Oksuz I, Xu H, Grau V, Guo F, Ng M, et al. Left Ventricle Quantification Challenge: A Comprehensive Comparison and Evaluation of Segmentation and Regression for Mid-Ventricular Short-Axis Cardiac MR Data. *IEEE Journal of Biomedical and Health Informatics* (2021) 25:3541–3553. doi:](http://paperpile.com/b/T2eW7l/YPEGN) [10.1109/jbhi.2021.3064353](http://dx.doi.org/10.1109/jbhi.2021.3064353)

219. [Zhuang X. Multivariate Mixture Model for Myocardial Segmentation Combining Multi-Source Images. *IEEE Trans Pattern Anal Mach Intell* (2019) 41:2933–2946.](http://paperpile.com/b/T2eW7l/9nZc5)

220. [Lalande A, Chen Z, Decourselle T, Qayyum A, Pommier T, Lorgis L, de la Rosa E, Cochet A, Cottin Y, Ginhac D, et al. Emidec: A Database Usable for the Automatic Evaluation of Myocardial Infarction from Delayed-Enhancement Cardiac MRI. *Brown Univ Dig Addict Theory Appl* (2020) 5:89.](http://paperpile.com/b/T2eW7l/fCKtr)

221. [Andreopoulos A, Tsotsos JK. Efficient and generalizable statistical models of shape and appearance for analysis of cardiac MRI. *Med Image Anal* (2008) 12:335–357.](http://paperpile.com/b/T2eW7l/y3WuL)

222. [Karim R, James Housden R, Balasubramaniam M, Chen Z, Perry D, Uddin A, Al-Beyatti Y, Palkhi E, Acheampong P, Obom S, et al. Evaluation of current algorithms for segmentation of scar tissue from late Gadolinium enhancement cardiovascular magnetic resonance of the left atrium: an open-access grand challenge. *Journal of Cardiovascular Magnetic Resonance* (2013) 15: doi:](http://paperpile.com/b/T2eW7l/Cfzt) [10.1186/1532-429x-15-105](http://dx.doi.org/10.1186/1532-429x-15-105)

223. [Tobon-Gomez C, Geers AJ, Peters J, Weese J, Pinto K, Karim R, Ammar M, Daoudi A, Margeta J, Sandoval Z, et al. Benchmark for Algorithms Segmenting the Left Atrium From 3D CT and MRI Datasets. *IEEE Trans Med Imaging* (2015) 34:1460–1473.](http://paperpile.com/b/T2eW7l/5DRU)

224. [Karim R, Blake L-E, Inoue J, Tao Q, Jia S, James Housden R, Bhagirath P, Duval J-L, Varela M, Behar JM, et al. Algorithms for left atrial wall segmentation and thickness – Evaluation on an open-source CT and MRI image database. *Medical Image Analysis* (2018) 50:36–53. doi:](http://paperpile.com/b/T2eW7l/ticn) [10.1016/j.media.2018.08.004](http://dx.doi.org/10.1016/j.media.2018.08.004)

225. [Corral Acero J, Xu H, Zacur E, Schneider JE, Lamata P, Bueno-Orovio A, Grau V. Left Ventricle Quantification with Cardiac MRI: Deep Learning Meets Statistical Models of Deformation. *Statistical Atlases and Computational Models of the Heart. Multi-Sequence CMR Segmentation, CRT-EPiggy and LV Full Quantification Challenges*. Springer International Publishing (2020). p. 384–394](http://paperpile.com/b/T2eW7l/pnco)

226. [Chen C, Liu Y, Schniter P, Tong M, Zareba K, Simonetti O, Potter L, Ahmad R. OCMR (v1.0)--Open-Access Multi-Coil k-Space Dataset for Cardiovascular Magnetic Resonance Imaging. *arXiv [eessIV]* (2020)](http://paperpile.com/b/T2eW7l/M9W8) <http://arxiv.org/abs/2008.03410>

227. [Moghari MH, Pace DF, Contreras H, Ghelani S, Olyaee E, Arasteh ST. *Whole-heart and Great Vessel Segmentation from 3D Cardiovascular Magnetic Resonance Images in Congenital Heart Disease (Part II)*. (2021). doi:](http://paperpile.com/b/T2eW7l/DaBZ) [10.5281/zenodo.4575238](http://dx.doi.org/10.5281/zenodo.4575238)

228. [Zhuang X, Li L, Wang S, Wu F. *Left Atrial and Scar Quantification & Segmentation Challenge 2022*. (2022). doi:](http://paperpile.com/b/T2eW7l/AN9i) [10.5281/zenodo.6362206](http://dx.doi.org/10.5281/zenodo.6362206)
